# Octopus-inspired deception and signaling systems from an exceptionally-stable acene variant

**DOI:** 10.1038/s41467-023-40163-7

**Published:** 2023-12-22

**Authors:** Preeta Pratakshya, Chengyi Xu, David J. Dibble, Aliya Mukazhanova, Panyiming Liu, Anthony M. Burke, Reina Kurakake, Robert Lopez, Philip R. Dennison, Sahar Sharifzadeh, Alon A. Gorodetsky

**Affiliations:** 1 Department of Chemistry, University of California, Irvine, Irvine, CA 92697 USA; 2 Department of Materials Science and Engineering, University of California, Irvine, Irvine, CA 92697 USA; 3 Department of Chemical and Biomolecular Engineering, University of California, Irvine, Irvine, CA 92697 USA; 4 Division of Materials Science and Engineering, Boston University, Boston, MA 02215 USA; 5 Department of Chemistry, Boston University, Boston, MA 02215 USA; 6 Department of Physics, Boston University, Boston, MA 02215 USA; 7 Department of Electrical and Computer Engineering, Boston University, Boston, MA 02215 USA

**Keywords:** Organic molecules in materials science, Organic molecules in materials science, Actuators

## Abstract

Multifunctional platforms that can dynamically modulate their color and appearance have attracted attention for applications as varied as displays, signaling, camouflage, anti-counterfeiting, sensing, biomedical imaging, energy conservation, and robotics. Within this context, the development of camouflage systems with tunable spectroscopic and fluorescent properties that span the ultraviolet, visible, and near-infrared spectral regions has remained exceedingly challenging because of frequently competing materials and device design requirements. Herein, we draw inspiration from the unique blue rings of the *Hapalochlaena lunulata* octopus for the development of deception and signaling systems that resolve these critical challenges. As the active material, our actuator-type systems incorporate a readily-prepared and easily-processable nonacene-like molecule with an ambient-atmosphere stability that exceeds the state-of-the-art for comparable acenes by orders of magnitude. Devices from this active material feature a powerful and unique combination of advantages, including straightforward benchtop fabrication, competitive baseline performance metrics, robustness during cycling with the capacity for autonomous self-repair, and multiple dynamic multispectral operating modes. When considered together, the described exciting discoveries point to new scientific and technological opportunities in the areas of functional organic materials, reconfigurable soft actuators, and adaptive photonic systems.

## Abstract (web summary)

The design of multifunctional platforms with tunable spectroscopic and fluorescent properties that span the ultraviolet, visible, and near-infrared spectral regions remains challenging. Here, the authors report an actuator-type system that leverages a readily-prepared, easily-processable, and exceptionally-stable nonacene-like molecule to achieve multiple complementary dynamic operating modes.

## Introduction

Multifunctional platforms that can adaptively modulate their color and appearance have attracted widespread attention for applications in displays, signaling, camouflage, anti-counterfeiting, sensing, biomedical imaging, energy conservation, and robotics^1–21^. Such technologically valuable platforms typically exhibit dynamic spectroscopic properties that emerge from programming of their constituent materials for responsiveness to specific external stimuli (e.g., mechanical, electrical, magnetic, chemical, or thermal inputs)^1–21^. Within this context, the development of systems with tunable spectroscopic and fluorescent properties that span the ultraviolet, visible, and near-infrared (UV–Vis–NIR) regions of the electromagnetic spectrum has proven challenging, especially for notoriously demanding tandem deception and signaling applications^1–11^. Indeed, relatively few classes of camouflage systems with the potential for achieving such desirable capabilities have been explored to date, including dual-band electrochromic devices, metamaterials/metasurfaces, electroactive polymers, reconfigurable photonic gels, and biomolecular films^1–11,22–30^. Despite much recent progress, these systems have all consistently struggled with multiple critical drawbacks, such as complex constituent active material preparation methodologies, sophisticated and expensive clean room fabrication schemes, difficult incorporation of more than one tunable spectroscopic capability, a lack of functionality across multiple spectral bands, the requirement for a single type of actuation strategy, slow switching and response times, degradation or inconsistent performance upon repeated actuation, and/or the inability to autonomously self-repair during operation without user intervention^1–11,22–30^. As such, there has emerged an exciting opportunity and powerful incentive for the development of multifunctional platforms that overcome the limitations of the reported technologies.

Within the realm of multifunctional deception and signaling platforms, one well-known class of active materials that has received comparatively minimal attention is acenes, which are organic polycyclic aromatic hydrocarbons consisting of linearly fused benzene rings and containing one aromatic sextet^31–50^. This relative lack of attention is surprising because acenes exhibit a variety of advantageous attributes, which include straightforward synthetic accessibility, chemically modular pi-conjugated core structures, amenability to peripheral substitution and functionalization, compatibility with bench-top fabrication and processing procedures, theoretically well-understood electronic characteristics, and stimuli-responsive spectroscopic properties^31–50^. Historically, the shorter acenes and heteroacenes (e.g., pentacene and its variants) have played pivotal roles in (1) organic chemistry as model polycyclic aromatic hydrocarbons (over the past ~70 years)^31–33,37,38^ and (2) organic optoelectronics as prototypical active materials for light-emitting diodes, solar cells, and field-effect transistors (over the past ~30 years)^42,43,50^. More recently, significant research effort has been expended on obtaining and characterizing longer acenes (and, in particular, nonacene derivatives) because of their significance from a fundamental physical organic chemistry viewpoint and their theoretically predicted superior properties for optoelectronic applications (e.g., smaller band gaps, higher mobilities, and tunable visible to near-infrared spectra)^33–36,39–41,44,48,51–61^. However, the solution-based synthesis of classic nonacene or its variants has been reported in a handful of studies (only 7 total), with these compounds featuring insufficient solid-state stabilities of at most two months even under inert conditions, qualitatively evaluated poor solution-phase stabilities of seconds to hours in ambient atmosphere under light, and limited solubilities in multiple kinds of solvent systems (Supplementary Table 1)^51–57^. Consequently, the previously studied nonacene variants could neither be processed into thin films nor used in conventional fabrication schemes and their utility for device applications was never explored (Supplementary Table 1)^51–61^, therefore leaving these molecules’ technological potential largely unknown.

**Fig. 1:**
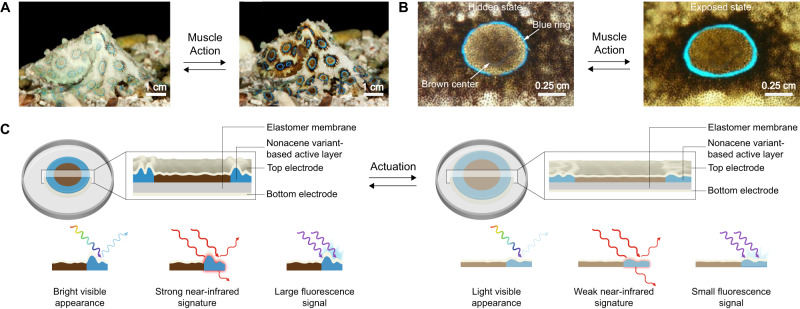
The blue-ringed octopus as biological inspiration and the conceptualization of deception and signaling systems. **A** Pictures of the *H. lunulata* octopus reversibly changing skin appearance when flashing its blue rings as a warning display^62^. **B** Pictures of an individual blue ring reversibly switching between hidden (left) and exposed (right) states on a contrast-enhancing brown dermal background^62^. **C** Schematics of the envisioned octopus-inspired deception and signaling systems before (left) and after (right) actuation. The proposed devices consist of a top transparent proton-conducting polymer electrode, a nonacene variant-based active layer wherein a wrinkled blue annulus surrounds a flatter brown circle, an underlying acrylic dielectric elastomer membrane, and a bottom transparent proton-conducting polymer electrode (top left and top right insets). Before actuation, the devices feature contracted microstructured active regions with bright visible appearances, strong near-infrared signatures, and large fluorescence signals (bottom left), but after actuation, the devices feature expanded nanostructured active regions with light visible appearances, weak near-infrared signatures, and small fluorescence signals (bottom right). The images in panels A and B are adapted from ref. ^62^ and reproduced with permission from The Company of Biologists Ltd.

Herein, we report bioinspired deception and signaling systems, which use a designer nonacene-like molecule with exceptional stability as the active material and thus demonstrate a unique combination of powerful capabilities. For the conceptualization of such systems, we examine the dynamic camouflage and aposematic warning display abilities of *Hapalochlaena lunulata* (*H. lunulata*) octopus skin (Fig. 1A), which are enabled by the muscle-controlled switching of iridescent blue rings between hidden and exposed states on a contrast-enhancing brown background (Fig. 1B)^62^. Moreover, we draw upon the literature precedent reported for the synthesis of nonacene derivatives^51–57^ and nitrogen-containing tetrabenzopentacenes^63,64^, as well as upon the technical foundation previously established in our studies of adaptive infrared camouflage systems^65–67^. Consequently, we envision blue ring-type dielectric elastomer actuator (DEA) devices consisting of a top transparent proton-conducting polymer electrode, a nonacene variant-based active layer wherein a wrinkled blue annulus surrounds a flatter brown circle, an underlying acrylic dielectric elastomer membrane, and a bottom transparent proton-conducting polymer electrode (Fig. 1C). Before actuation, we anticipate that such devices will feature contracted microstructured active regions with bright visible appearances, strong associated near-infrared signatures, and large corresponding fluorescence signals (Fig. 1C, left), but after actuation, we anticipate that such devices will feature expanded nanostructured active regions with lighter visible appearances, weaker associated near-infrared signatures, and smaller corresponding fluorescence signals (Fig. 1C, right). Due to this design strategy, we envisage that the proposed deception and signaling devices will demonstrate functionalities analogous to those of the *H. lunulata* octopus’ blue rings and exhibit a highly desirable combination of capabilities and metrics not reported together for any appearance-changing platform to date^1–11^.

## Results

### Guided design, facile synthesis, and computational analysis of the nonacene-like molecule

**Fig. 2:**
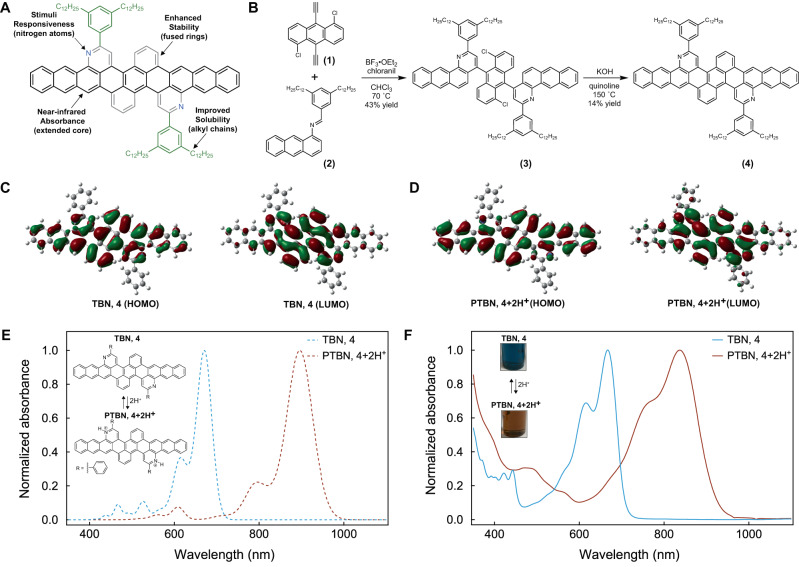
Design, synthesis, simulation, and spectroscopy of the nonacene variant. **A** The design of our nonacene-like molecule, where the extended all-carbon nonacene core (black) results in a near-infrared absorbance, the fused peripheral aromatic rings (gray) enhance stability, the nitrogen heteroatoms (blue) introduce stimuli responsiveness, and the pendant alkyl-functionalized phenyl rings (green) improve solubility. **B** The synthetic route for the preparation of nonacene-like molecule **4**, where the two key steps involve a Lewis acid-mediated aza-Diels-Alder reaction and a base-mediated cyclodehydrohalogenation reaction. **C** The HOMO (left) and LUMO (right) calculated for the unprotonated tetrabenzononacene TBN, **4**. **D** The HOMO (left) and LUMO (right) calculated for PTBN, **4** + **2H**^**+**^. **E** The theoretical UV–Vis–NIR absorption spectra calculated for TBN, **4** (blue dashed line) and PTBN, **4** + **2H**^**+**^ (brown dashed line). The chemical structures of TBN and PTBN are inset. **F** The experimental UV–Vis–NIR absorption spectra measured for TBN, **4** (blue solid line) and PTBN, **4** + **2H**^**+**^ (brown solid line). The pictures of the TBN and PTBN solutions in chloroform are inset. Note that the theoretical and experimental absorption spectra were normalized to unity for clarity.

As the active material for our bioinspired platform, we conceived and designed a nonacene-like molecule with a unique architecture by drawing upon literature precedent. In our design, we first started with an extended all-carbon nonacene core, i.e., nine ortho-annulated benzene rings in a linear arrangement, which would furnish a relatively low bandgap and thus a red-to-near-infrared absorbance (Fig. 2A, black)^33,34^. We next expanded this pi-conjugated core by adding four peripheral fused aromatic rings, which would substantially enhance stability through introduction of multiple aromatic sextets (Fig. 2A, gray)^53,55^. We in turn incorporated nitrogen heteroatoms within two of the peri-annulated rings, which would afford responsiveness to chemical stimuli such as protonation (Fig. 2A, blue)^47,49^. We last included pendant alkyl chain-functionalized phenyl rings near the nitrogens, which would mitigate intramolecular pi-pi stacking and improve solubility in multiple different solvents (Fig. 2A, green)^31,51^. We theorized that the resulting nonacene-like molecular architecture would feature the spectroscopic properties, ambient-atmosphere stability, and general good solubility essential for demanding camouflage (or other) device applications.

We initially developed a facile strategy for the synthesis of our nonacene-like molecule, which was denoted as tetrabenzononacene or TBN in accordance with prior precedent^63,64^. For this purpose, we employed modified versions of the literature methodologies established for the preparation and characterization of nitrogen-containing tetrabenzopentacenes, polydiquinolineanthracenes, and zigzag polyquinolines (see sections I.A.1 and I.A.2 in the Supplementary Information)^63,64,68,69^. In brief, we initially obtained the previously reported 1,5-dichloro-9,10-diethynylanthracene (**1**) precursor and the required didodecyl-substituted phenylmethanimine anthracene (**2**) precursor according to known procedures (see section I.A.2 in the Supplementary Information and Fig. 2B, left)^63,64,68,69^. We then generated the key dinaphthoquinolineanthracene (**3**) intermediate in reasonable yield by reacting precursors (**1**) and (**2**) via the Lewis acid-mediated aza-Diels–Alder (Povarov) reaction (see section I.A.2 in the Supplementary Information and Fig. 2B, middle)^63,64,68,69^. We next converted intermediate (**3**) into the desired nonacene-like molecule (**4**) in moderate yield by forming internal C–C bonds between the anthracene core and its two pendant naphthoquinolines via a base-mediated cyclodehydrohalogenation reaction (see section I.A.2 in the Supplementary Information and Fig. 2B, right)^63^. Here, we characterized precursor (**2**), intermediate (**3**), and product (**4**) via standard methods, i.e., a combination of ^1^H NMR spectroscopy, ^13^C NMR spectroscopy, and mass spectrometry (see Supplementary Figs. 1–8 and section I.A.3 in the Supplementary Information). In addition, we further confirmed the identity of product (**4**) by performing size exclusion chromatography (SEC) for the isolated pure material (Supplementary Fig. 9), simulating the obtained matrix-assisted laser-desorption ionization (MALDI) mass spectrum to confirm the isotopic distribution pattern (Supplementary Figure 10), and collecting 2D ^1^H–^1^H correlation spectroscopy (COSY) spectra to assign the expected multi-nuclear spin systems (Supplementary Figure 11). Overall, our target compound was synthesized by employing straightforward reaction conditions, isolated in yields reasonable for quantities of tens-to-hundreds of milligrams, and rigorously characterized with a suite of complementary techniques.

We proceeded to obtain computational insight into the likely conformation and electronic properties of our synthesized nonacene-like molecule. Specifically, we calculated the ground-state geometries, electronic structures, and molecular orbitals for both the unprotonated tetrabenzononacene (TBN, **4**) and the protonated tetrabenzononacene (PTBN, **4** + **2H**^**+**^) by employing conventional density functional theory (DFT) and unrestricted broken symmetry DFT with the B3LYP functional and the 6-311G (d,p) basis set (note that the side chains were excluded for computational tractability) (see section I.B.1 in the Supplementary Information)^53–55,63,70–75^. The molecular geometries predicted for TBN and PTBN by using standard DFT revealed twisted concave nonacene cores and downward-oriented tilted phenyl rings, suggesting that solubility would be enhanced due to disruption of intermolecular pi–pi stacking interactions (see Supplementary Data 1 and Supplementary Data 2). The corresponding highest occupied molecular orbitals (HOMOs) and lowest unoccupied molecular orbitals (LUMOs) calculated for TBN and PTBN by using standard DFT featured comparable shapes and nearly encompassed the pi-conjugated cores, albeit with reduced LUMO electron densities at the molecular termini (Fig. 2C, D). Here, the HOMO and LUMO energies were –5.01 eV and –3.11 eV, respectively, for TBN, whereas the HOMO and LUMO energies were –9.59 eV and –8.07 eV, respectively, for PTBN, suggesting enhanced oxidation resistance for the molecule upon protonation^37–39,44,45,49^. Moreover, the electronic configurations predicted for TBN and PTBN with unrestricted broken symmetry DFT were closed-shell, suggesting reduced reactivity for the molecule with respect to the open-shell all-carbon nonacene (Supplementary Table 1)^51–58,74,75^. The calculations and analyses together indicated that our designer macromolecule would possess increased solubility in standard solvents and enhanced stability in ambient atmosphere.

We also obtained computational insight into the anticipated optical properties of our synthesized nonacene-like molecule. In particular, we simulated the ultraviolet–visible–near-infrared (UV–Vis–NIR) absorption spectra for TBN and PTBN by employing time-dependent density functional theory (TDDFT) together with the Franck-Condon approximation and evaluated the nature of the associated electronic transitions by leveraging procedures validated for analogous molecular systems (see section I.B.2 in the Supplementary Information)^72,73,76–81^. For TBN (Fig. 2E, inset), the simulated absorption spectrum revealed an onset at ~725 nm, a main peak at ~670 nm resulting from the first two excited states, a large shoulder at ~632 nm resulting from the vibronic progression of the two lower-energy excited states, and multiple smaller peaks between ~550 nm and ~420 nm corresponding to the three higher-energy excited states (Fig. 2E, blue and Supplementary Fig. 12). For PTBN (Fig. 2E, inset), the simulated absorption spectrum revealed an onset at ~1000 nm, a main peak at ~896 nm resulting from the two lower-energy excited states, a large shoulder at ~798 nm resulting from the vibronic progression of the first two excited states, and multiple smaller peaks between ~650 nm and ~520 nm corresponding to the three higher-energy excited states (Fig. 2E, brown and Supplementary Fig. 13). Here, the absorption spectrum predicted for PTBN was red-shifted by >200 nm relative to the one predicted for TBN, in agreement with theoretical and experimental precedent for N-heteroacenes^47,49,72,73^. Interestingly, the bright first excited states and dark second excited states calculated for TBN and PTBN were polarized along and orthogonal to the nonacene core, in contrast to computational findings for the parent all-carbon molecule (Supplementary Table 2)^80,81^. The simulations and analyses together indicated that our designer macromolecule would absorb light in the red-to-near-infrared regions of the electromagnetic spectrum and that a routine chemical stimulus (protonation) would shift its absorbance further into the near-infrared range.

### Solution-phase and solid-state spectroscopic characterization of the nonacene-like molecule

We further advanced our efforts by systematically investigating the electronic and optical properties of our nonacene-like molecule. For this purpose, we prepared TBN solutions by dissolving the compound in organic solvents, converted TBN solutions into PTBN solutions through the addition of acid, and interrogated such solutions with UV–Vis–NIR spectroscopy in ambient atmosphere (see sections I.C.1 and I.C.2 in the Supplementary Information). For TBN solubilized in chloroform, the absorption spectra revealed an onset at ~708 nm, a major peak with a maximum at ~667 nm, and a cluster of sharp small peaks between ~600 nm and ~360 nm (Fig. 2F, blue). Such solutions featured a bright blue color at high molecular concentrations (Fig. 2F, inset). For PTBN solubilized in chloroform, the absorption spectra revealed an onset of ~924 nm, a major peak with a maximum at ~837 nm, and a cluster of broadened small peaks between ~600 nm and ~400 nm (Fig. 2F, brown). Such solutions featured a dark brown color at high molecular concentrations (Fig. 2F, inset). Here, the absorption spectra obtained for PTBN were red-shifted by >170 nm relative to those obtained for TBN, as expected from our theoretical predictions (vide supra) and extensive literature precedent for N-heteroacenes^47,49,72,73^. Moreover, the conversion of TBN into PTBN was dependent on the amount of added acid, reversible with concentrated base, and achievable in multiple solvent/acid systems (Supplementary Figs. 14, 15). Notably, the experimentally obtained spectra were in reasonable agreement with the theoretically predicted spectra, with the differences likely resulting from the limited accuracy of the employed functionals and the influence of challenging-to-simulate solvation effects (compare, for example, Fig. 2E, F and Supplementary Figs. 14, 15)^82,83^. These spectroscopic measurements demonstrated that our designer macromolecule featured good solubility and proved that protonation could modulate its visible to near-infrared solution absorbance.

We next evaluated the stability of our nonacene-like molecule under conditions that would prove challenging for any acene variant. Towards this end, we prepared solutions containing TBN, PTBN, or 6,13-bis((trimethylsilyl)ethynyl)pentacene (i.e., TMS-pentacene, which is a classic reference compound with well-known improved stability)^45,84^, and we then comparatively characterized such solutions with UV–Vis–NIR spectroscopy during high-intensity broadband illumination in ambient atmosphere (see section I.C.3 in the Supplementary Information). Under our intentionally harsh conditions, TBN’s peak absorbance decayed relatively rapidly with a half-life of 0.3 ± 0.1 minutes, but PTBN’s peak absorbance decayed > 7800-fold more slowly with a half-life of 2358 ± 307 min (Supplementary Fig. 16). By comparison, TMS-pentacene’s peak absorbance decayed rapidly with a half-life of 35.2 ± 1.5 min (Supplementary Fig. 16). Therefore, both TBN and PTBN withstood environmental conditions that would instantaneously decompose any of the reported nonacenes (Supplementary Table 1), with PTBN even exhibiting a >65-fold solution-phase stability enhancement relative to the well-known TMS-pentacene reference compound. Remarkably, TBN showed no obvious signs of degradation even after >2 years of storage as a solid or in solution and thus demonstrated a >10-fold stability enhancement relative to the reported nonacene derivatives, as confirmed by UV–Vis–NIR spectroscopy, proton NMR spectroscopy, and MALDI mass spectrometry (see Supplementary Table 1, section I.A.2 in the Supplementary Information, and Supplementary Figs. 7, 11, 17, 18). These measurements showcased our designer macromolecule’s outstanding general robustness under extreme conditions and therefore portended favorably for the material’s utility in demanding deception and signaling applications.

**Fig. 3:**
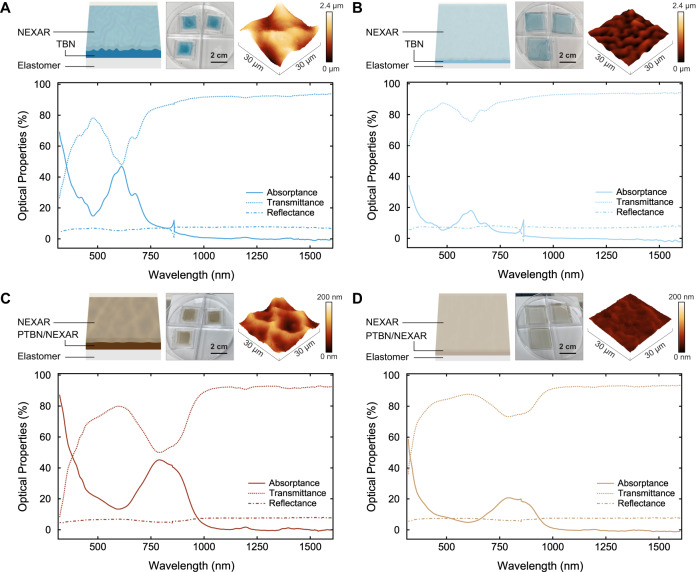
Physical and spectroscopic characterization of films from TBN and PTBN in device-relevant configurations. **A** Top: Schematic (left), pictures (middle), and AFM images (right) of unactuated TBN-based tri-layer architectures. Bottom: Representative UV–Vis–NIR absorptance (dark blue solid line), transmittance (dark blue dotted line), and reflectance (dark blue dashed-dotted line) spectra for unactuated tri-layer TBN-based architectures. **B** Top: Schematic (left), pictures (middle), and AFM images (right) of actuated TBN-based tri-layer architectures. Bottom: Representative UV–Vis–NIR absorptance (light blue solid line), transmittance (light blue dotted line), and reflectance (light blue dashed-dotted line) spectra for actuated TBN-based tri-layer architectures. **C** Top: Schematic (left), pictures (middle), and AFM images (right) of unactuated PTBN-based tri-layer architectures. Bottom: Representative UV–Vis–NIR absorptance (dark brown solid line), transmittance (dark brown dotted line), and reflectance (dark brown dashed-dotted line) spectra for unactuated PTBN-based tri-layer architectures. **D** Top: Schematic (left), pictures (middle), and AFM images (right) of actuated PTBN-based tri-layer architectures. Bottom: Representative UV–Vis–NIR absorptance (light brown solid line), transmittance (light brown dotted line) and reflectance (light brown dashed-dotted line) spectra for actuated PTBN-based tri-layer architectures.

We in turn studied the solid-state spectroscopic properties of films from highly soluble, exceptionally stable, and readily processable TBN in a device-relevant configuration. To achieve this goal, we fabricated tri-layer architectures for which a dropcast neat TBN film was the central layer by drawing upon protocols validated for analogous systems^66,67^, and we characterized these architectures with digital camera imaging (DCI), atomic force microscopy (AFM), and UV–Vis–NIR spectroscopy, both without and with mechanical actuation (see Supplementary Fig. 19A and section I.D in the Supplementary Information). The unactuated TBN-based architectures featured bright blue colorations and microstructured surface topographies (Fig. 3A, top). The corresponding spectra revealed absorptances of ~30.6 ± 2.6%, transmittances of ~63.4 ± 2.5%, and reflectances of ~6.0 ± 0.1% at ~676 nm (Fig. 3A, bottom). The actuated TBN-based architectures featured lighter blue colorations and >2-fold flatter topographies (Fig. 3B, top). The corresponding spectra revealed decreased absorptances of ~11.6 ± 1.7%, increased transmittances of ~81.1 ± 1.1%, and comparable reflectances of ~7.2 ± 0.6% at ~676 nm (Fig. 3B, bottom). Here, our TBN-based architectures’ lowered absorptances and enhanced transmittances presumably resulted from strain-induced reductions in both their effective thicknesses and substantial surface roughnesses, in agreement with prior findings^66,67^. Remarkably, TBN-based architectures fabricated from TBN solutions stored for >2 years featured actuation-dependent visible appearances, surface topologies, and spectroscopic characteristics that were quite similar to those of analogous architectures fabricated from freshly prepared TBN solutions (compare Fig. 3A, B and Supplementary Fig. 20). The experiments indicated that our nonacene-like molecule was a suitable active material for the envisioned deception and signaling platforms.

We analogously studied the solid-state spectroscopic properties of films from highly soluble, exceptionally stable, and readily processable PTBN in a device-relevant configuration. To achieve this goal, we fabricated tri-layer architectures for which a dropcast mixed PTBN**/**NEXAR^TM^ sulfonated pentablock copolymer (NEXAR) film was the central layer by drawing upon protocols validated for analogous systems^66,67^, and we characterized the architectures with DCI, AFM, and UV–Vis–NIR spectroscopy, both without and with mechanical actuation (see Supplementary Fig. 19B and section I.D in the Supplementary Information). The unactuated PTBN-based architectures featured dark brown colorations and nanostructured surface topographies (Fig. 3C, top). The corresponding spectra revealed absorptances of ~43.7 ± 1.2%, transmittances of ~51.3 ± 1.1%, and reflectances of ~5.0 ± 0.1% at ~792 nm (Fig. 3C, bottom). The actuated PTBN-based architectures featured lighter brown colorations and >3-fold flatter surface topographies (Fig. 3D, top). The corresponding spectra revealed decreased absorptances of ~21.4 ± 2.4%, increased transmittances of ~72.5 ± 2.3%, and comparable reflectances of ~6.1 ± 0.2% at ~792 nm (Fig. 3D, bottom). Here, our PTBN-based architectures’ lowered absorptances and enhanced transmittances presumably resulted primarily from a strain-induced reduction in their effective thicknesses, in agreement with prior findings^66,67^. The experiments further underscored the suitability of our nonacene-like molecule for applications in the envisioned deception and signaling platforms.

### Development and validation of deception and signaling systems from the nonacene-like molecule

**Fig. 4:**
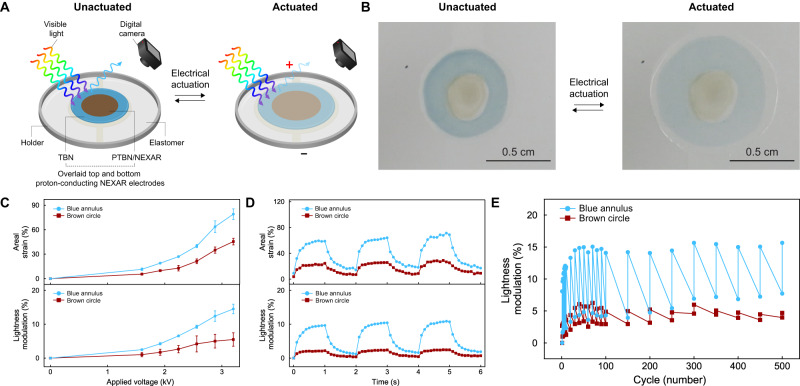
Appearance-changing functionalities, operational performances, and cycling stabilities of TBN- and PTBN-based systems. **A** Schematic of a visible appearance-changing system undergoing monitoring during electrical actuation under standard indoor lighting in ambient atmosphere. **B** Representative digital camera images of an appearance-changing device before (left) and after (right) electrical actuation with a bias of ~3.2 kV. Note the change in area and color lightness for the blue-brown annulated circle as a result of electrical actuation. **C** Top: Plot of the areal strain as a function of the applied voltage for the devices’ outer annuli (blue line) and inner circles (brown line). Bottom: Plot of the lightness modulation as a function of the applied voltage for the devices’ outer annuli (blue line) and inner circles (brown line). The error bars represent standard deviations. **D** Top: Plot of the areal strain as a function of time for a representative device’s outer annulus (blue line) and inner circle (brown line) over three consecutive on/off actuation cycles. Bottom: Plot of the lightness modulation as a function of time for a representative device’s outer annulus (blue line) and inner circle (brown line) over three consecutive on/off actuation cycles. **E** Plot of the lightness modulation as a function of the actuation cycle number for a representative device’s outer annulus (blue line) and inner circle (brown line) over 500 sequential actuation cycles.

We continued our efforts by manufacturing the envisioned octopus blue ring-inspired systems and studying their appearance-changing functionalities within the visible region of the electromagnetic spectrum. For this purpose, we employed modified and improved versions of methodologies previously established for the preparation of adaptive infrared camouflage platforms with less advanced capabilities^66,67^. We accordingly fabricated quad-layer dielectric elastomer actuator (DEA) devices for which the active layer consisted of TBN-based blue annulus enclosing a PTBN/NEXAR-based brown circle (see Supplementary Fig. 21 and section I.E.1 in the Supplementary Information). We then visualized and quantified the color lightness and areal strain for such DEA devices via DCI during variable-bias electrical actuation above a white scattering background under standard indoor lighting in ambient atmosphere (see sections I.E.2 and I.E.3 in the Supplementary Information). For our devices, the blue-brown annulated circles simultaneously expanded laterally and became noticeably lighter upon actuation with large biases of either ~2.9 kV or ~3.2 kV (Supplementary Fig. 22, Fig. 4A, B, and Supplementary Movie 1). The active regions’ areal strains and lightness changes featured characteristic dependences on the magnitude of the applied voltage, with average strains of ~79 ± 6% and ~45 ± 4% and average lightness changes of ~14 ± 1% and ~5 ± 2% for the blue outer annuli and brown inner circles, respectively, at a ~3.2 kV bias (Fig. 4C). Moreover, the devices exhibited rapid response times of ~380 msec when repeatedly actuated by a 0.5 Hz frequency square waveform with a 0 kV minimum and a ~2.9 kV maximum (Fig. 4D). When considered together, the experiments showed that devices from our nonacene-like molecule featured dynamic visible appearance-changing capabilities and operational performances that compared favorably to those reported for analogous camouflage platforms^1–11,65–67^.

Having demonstrated the appearance-changing functionalities of our octopus blue ring-inspired systems, we sought to rigorously evaluate their operational stabilities. To achieve this goal, we systematically characterized the color lightness of 10 different quad-layer DEA devices via DCI during hundreds of rounds of repeated electrical actuation with a ~2.9 kV bias, again above a white scattering background under standard indoor lighting in ambient atmosphere (see sections I.E.2 and I.E.3 in the Supplementary Information). For 7 of the 10 devices, the blue-brown annulated circles featured uniform coloration and revealed no obvious evidence of physical delamination or electrochemical degradation (Supplementary Fig. 22). These 7 devices continuously operated with stable lightness modulation over at least 500 cycles (Fig. 4E and Supplementary Table 3). For 3 of the 10 devices, the blue-brown annulated circles initially featured uniform coloration but then acquired localized discoloration suggestive of chemical degradation (Supplementary Fig. 23). These 3 devices continuously operated with stable lightness modulation for ~100 to ~400 cycles, spontaneously failed with a corresponding brief pause in functionality, and resumed operating but with altered lightness modulation for ~50 to ~100 more cycles (Supplementary Fig. 24 and Supplementary Table 3). Here, our devices presumably failed through classical dielectric breakdown and pinhole shorting mechanisms common for dielectric elastomer actuators (and other capacitors) and then likely self-repaired through local conversion of our nonacene-like molecule and/or the acrylic elastomer into an insulating material (see Supplementary Fig. 23)^85–87^. These experiments highlighted our devices’ exceptional ambient atmosphere cycling stabilities and revealed their ability to autonomously self-repair without any user intervention, which is a rare capability among dielectric elastomer actuators^88,89^ and has not been reported for analogous camouflage platforms^1–11,65–67^.

**Fig. 5:**
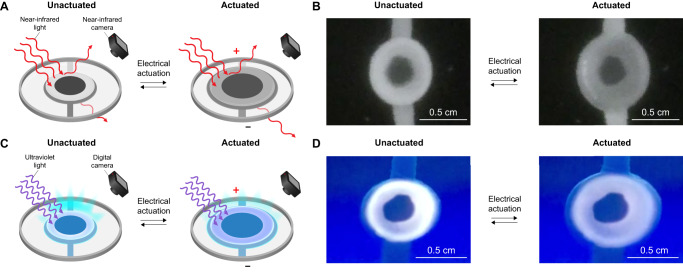
Signature management and signaling functionalities of TBN- and PTBN-based systems. **A** Schematic of a near-infrared signature management system undergoing monitoring during electrical actuation under near-infrared illumination in ambient atmosphere. **B** Representative spectrally filtered digital camera images of a signature management device before (left) and after (right) electrical actuation with a bias of ~3.2 kV. Note the changes in area and near-infrared contrast for the annulated circle as a result of electrical actuation. **C** Schematic of a fluorescence signaling system undergoing monitoring during electrical actuation under ultraviolet illumination in ambient atmosphere. **D** Representative digital camera images of a signaling device before (left) and after (right) electrical actuation with a bias of ~3.2 kV. Note the changes in area and fluorescence signal intensity for the annulated circle as a result of electrical actuation.

We subsequently investigated the signature management functionalities of our octopus blue ring-inspired systems within the near-infrared region of the electromagnetic spectrum. Towards this end, we visualized and quantified the near-infrared contrast and areal strain for quad-layer devices via spectrally filtered DCI during electrical actuation above a black absorbing background under near-infrared illumination in ambient atmosphere (see section I.E.4 in the Supplementary Information). For our devices, the annulated circles expanded laterally and decreased in contrast with respect to the surroundings upon actuation with a large bias of ~3.2 kV (Fig. 5A, B). The active regions’ outer annuli and inner circles featured average areal strains of ~73 ± 3% and ~46 ± 2% and average contrast changes of ~–27 ± 3% and ~–19 ± 5%, respectively, at a ~3.2 kV bias (Fig. 5A, B). The devices moreover demonstrated robust and consistent near-infrared contrast changes during multiple sequential actuation cycles (Supplementary Movie 2). Here, the contrast switching observed for the TBN-based outer annuli and PTBN-based inner circles presumably resulted primarily from modulation of their surface roughness-induced scattering and near-infrared absorbances (see Fig. 3)^66,67,90^. These experiments showed that devices from our nonacene-like molecule possess near-infrared deception capabilities, which remain highly valued and sought after in camouflage applications^1–11,65–67^.

We last investigated the signaling functionalities of our octopus blue ring-inspired systems within the visible to near-infrared regions of the electromagnetic spectrum. Towards this end, we visualized and quantified the fluorescence signal intensity and areal strain for quad-layer devices via DCI during electrical actuation above a white scattering background under ultraviolet illumination in ambient atmosphere (see section I.E.5 in the Supplementary Information). For our devices, the annulated circles expanded laterally and decreased in signal intensity with respect to the surroundings upon actuation with a large bias of ~3.2 kV (Fig. 5C, D). The active regions’ outer annuli and inner circles featured average areal strains of ~74 ± 4% and ~43 ± 4% and average signal intensity changes of ~–18 ± 1% and ~–6 ± 2%, respectively, at a ~3.2 kV bias (Fig. 5C, D). The devices moreover demonstrated robust and consistent fluorescence signal intensity changes during multiple sequential actuation cycles (Supplementary Movie 3). Here, the TBN-based outer annuli exhibited larger signal intensity switching with respect to the PTBN-based inner circles because TBN featured a much stronger visible to near-infrared fluorescence relative to PTBN under ultraviolet (or other) excitation (see Supplementary Fig. 25). These experiments showed that devices from our nonacene-like molecule not only operate stably but also exhibit desirable multispectral signaling capabilities, even under challenging and deleterious continuous ultraviolet irradiation.

## Discussion

In summary, we have reported a readily synthesized, theoretically tractable, highly soluble, and exceptionally stable nonacene-like molecule, and our discoveries represent an exciting advance from an organic materials chemistry perspective for multiple reasons. First, our functionalized and expanded nonacene-like molecule is synthesized by employing straightforward reaction conditions and in yields reasonable for quantities of tens-to-hundreds of milligrams, thereby adding to the few previously described solution-phase syntheses of comparable large acenes (Supplementary Table 1). Additionally, our nonacene-like molecule features tunable electronic, optical, and physical properties that are inspired by literature precedent and are in good agreement with theoretical predictions (vide supra), thus establishing a conceptual framework for the continued engineering and improvement of its highly desirable characteristics. Furthermore, the molecule demonstrates unrivaled and exceptional stability, including the ability to survive direct high-intensity illumination for over a full day when protonated (representing a >65-fold improvement relative to a well-known acene standard) and the ability to withstand solution-phase or solid-state storage for >2 years with no obvious degradation (representing a >10-fold improvement relative to the reported nonacene derivatives) (Supplementary Table 1). Last, the molecule exhibits good solubility at high concentrations in multiple different solvents, presumably due to its twisted aromatic core and pendant alkyl chain-functionalized phenyl rings, and thus can be directly processed into large-area films via standard techniques even after >2 years of storage, which has not been possible for other nonacenes (Supplementary Table 1). Overall, the described designer macromolecule features an outstanding combination of characteristics that objectively improve upon and advance the comparable state-of-the-art acene materials.

We have moreover taken advantage of our nonacene-like molecule’s favorable physical properties for the development of multifunctional octopus-inspired deception and signaling systems, and our discoveries are exciting from the perspective of color- and appearance-changing platforms for multiple reasons. First, appearance-changing systems from our nonacene-like molecule are fabricated via routine benchtop techniques using minimal equipment and constitute the only reported integration of a nonacene variant into functional devices of any kind (Supplementary Table 1), thereby reinforcing the exceptional stability and ready processability of our active material. Additionally, the baseline performance of our electromechanical systems compares favorably to that reported for analogous camouflage platforms, with figures of merit that include maximum areal strains >~90% and response times of <~400 ms. Furthermore, our actuator-type systems not only consistently and reliably change their visible appearance for ~500 cycles, with minimal-to-no degradation in functionality under ambient conditions, but also unexpectedly possess the ability to autonomously self-repair without user intervention (Supplementary Table 3), which is rare among other dielectric elastomer actuators and not known for analogous camouflage platforms. Last, our systems demonstrate a unique combination of capabilities in the UV–Vis–NIR region of the electromagnetic spectrum, including the ability to modulate visible color lightness, change near-infrared contrast, and adjust multispectral fluorescence intensity. Altogether, our octopus-inspired deception and signaling systems feature a powerful and unique combination of advantages with respect to other camouflage technologies (e.g., straightforward benchtop fabrication, competitive baseline performance metrics, robustness during cycling with the capacity for autonomous self-repair, and multiple dynamic multispectral operating modes)^1–11,65–67^.

When looking forward, we anticipate that our discoveries will afford a range of promising scientific and technological opportunities for both organic electronic materials and appearance-changing platforms alike. For example, our computationally validated molecular engineering strategy and facile solution-phase synthetic methodology could be extended to the preparation of longer variably functionalized acenes, allowing for the fundamental investigation of these molecules’ length-dependent electronic structures and, most critically, anticipated ambient-atmosphere stabilities. In turn, the advantageous photophysical robustness and solution-phase processability of our nonacene-like molecule and, presumably, of its longer variants, will facilitate future investigation of such materials within the context of traditional optoelectronic systems (e.g., light-emitting diodes, solar cells, and field effect transistors), therefore allowing for the elucidation of extended acenes’ theoretically predicted but experimentally elusive superior materials properties for traditional devices. Subsequently, the routine benchtop strategies validated for the fabrication of our octopus-inspired deception and signaling systems, together with the favorable physical properties expected for longer analogous designer acene variants, should enable the scalable manufacturing of devices with even larger areas, as well as the incorporation of additional dynamic modalities for such devices, thereby addressing other grand challenges for all camouflage technologies. Finally, the further detailed exploration of our systems’ long-term cyclical stabilities and unexpected autonomous self-repair capabilities may unlock new approaches to understanding and mitigating classical defect-based failure mechanisms for both dielectric elastomer actuators and color- and appearance-changing platforms. Assuming the realization of the described possibilities, the exciting findings reported herein could ultimately influence areas as varied as organic and functional materials, bioinspired and biomimetic photonics, biological and chemical sensing, biomedical imaging and bioelectronics, energy generation and conservation, and soft actuation and robotics.

## Methods

The extended details for the Synthesis and Chemical Characterization of the Nonacene-Like Molecule are provided in Section I.A of the Supplementary Information; the extended details for the Computational Analysis of the Nonacene-Like Molecule are provided in Section I.B of the Supplementary Information; the extended details for the Solution-Phase Spectroscopy of the Nonacene-Like Molecule are provided in Section I.C of the Supplementary Information; the extended details for the Fabrication, Mechanical Actuation, and Characterization of the Tri-Layer Architectures from the Nonacene-Like Molecule are provided in Section I.D of the Supplementary Information; and the extended details for the Fabrication, Electrical Actuation, and Characterization of the Quad-Layer Devices from the Nonacene-Like Molecule are provided in Section I.E of the Supplementary Information.

### Supplementary information

Supplementary Information

Description of Additional Supplementary Files

Supplementary Data 1

Supplementary Data 2

Supplementary Movie 1

Supplementary Movie 2

Supplementary Movie 3

### Source data

Source Data

## Data Availability

All data needed to evaluate the described conclusions are present in the manuscript and/or the supplementary materials. The coordinates of the optimized structures for TBN, **4** and PTBN, **4** + **2H**^**+**^ are provided in the source data files. Other relevant data are available from the authors upon request. Source data are provided with this paper.
