# Supplementary material for: Octopus-inspired deception and signaling systems from an exceptionally-stable acene variant
Source: Nat Commun. 2023 Dec 22;14:8528. doi: 10.1038/s41467-023-40163-7 (PMC10746719; doi:10.1038/s41467-023-40163-7)
Supplement: Supplementary file 1 — Supplementary Information [file 41467_2023_40163_MOESM1_ESM.pdf]

Supplementary Information

for

Octopus-inspired deception and signaling systems from an exceptionally-stable acene variant

Preeta Pratakshya,[1,8] Chengyi Xu,[2,8] David J. Dibble,[3,8] Aliya Mukazhanova,[4] Panyiming Liu,[2] Anthony M. Burke,[3] Reina Kurakake,[2] Robert Lopez,[2] Philip R. Dennison,[1] Sahar Sharifzadeh,[4,5,6,7] Alon A. Gorodetsky[2,3*]

[1]Department of Chemistry, University of California, Irvine, Irvine, CA 92697, USA

[2]Department of Materials Science and Engineering, University of California, Irvine, Irvine, CA 92697, USA

[3]Department of Chemical and Biomolecular Engineering, University of California, Irvine, Irvine, CA 92697, USA

[4]Division of Materials Science and Engineering, Boston University, Boston, MA 02215, USA

[5]Department of Chemistry, Boston University, Boston, MA 02215, USA

[6]Department of Physics, Boston University, Boston, MA 02215, USA

[7]Department of Electrical and Computer Engineering, Boston University, Boston, MA 02215, USA

[8]These authors contributed equally: Preeta Pratakshya, Chengyi Xu, David J. Dibble

*E-mail to alon.gorodetsky@uci.edu

I. Supplementary Methods

A. Synthesis and Chemical Characterization of the Nonacene-Like Molecule.

1. Materials. All chemicals, solvents, and supplies were purchased from Thermo Fisher Scientific, Teledyne Isco, Sigma Aldrich, Acros Organics, Aurum Pharmatech, Combi-Blocks, Univar Solutions, Gallade Chemical, or Kraton Polymers. Typically, toluene, chloroform, and other solvents were dried with 3 Å molecular sieves and stored under argon. Unless otherwise noted, the glassware was oven dried at 150 ºC – 200 ºC, and the reactions were performed under dry argon. When necessary, the silica gel columns/cartridges used for purification were flushed with 1/9 triethylamine/hexanes to deactivate the gel and with hexanes to remove excess triethylamine.

2. Synthetic Protocols.

a. *N*-(3,5-didodecylbenzylidene)anthracene-1-amine (2). This precursor was typically prepared according to modified versions of established procedures.[1-4] First, a mixture of commercially-available 1-anthyrylamine (2.88 g, 14.9 mmol), previously-reported 3,5-didodecylbenzaldehyde (6.00 g, 13.6 mmol),[2,3] glacial acetic acid (~ 0.1 mL), and molecular sieves (10.0 g) in toluene (200 mL) was combined in a sealed pressure vessel and stirred under argon at 150 ºC for 5 days. Next, the reaction mixture was cooled to room temperature and filtered through Celite. Subsequently, the solvent was removed *in vacuo.* Last, the crude material was dissolved in chloroform and isolated by trituration from methanol to yield a brown oil (6.80 g, 81% yield): ^{1}H NMR (500 MHz, $CDCl_3$) δ 8.88 (s, 1H), 8.58 (s, 1H), 8.43 (s, 1H), 8.06 – 8.00 (m, 2H), 7.88 (d, *J* = 8.5 Hz, 1H), 7.71 (d, *J* = 1.6 Hz, 2H), 7.51 – 7.43 (m, 3H), 7.20 (t, *J* = 1.7 Hz, 1H), 6.98 (d, *J* = 6.9 Hz, 1H), 2.71 (t, *J* = 7.8 Hz, 4H), 1.71 (quint, *J* = 7.5 Hz, 4H), 1.42 – 1.26 (m, 36H), 0.88 (t, *J* = 6.9 Hz, 6H); ^{13}C NMR (126 MHz, $CDCl_3$) δ 161.26, 150.24, 143.80, 135.20, 132.32, 132.22,

131.67, 128.94, 128.09, 127.83, 127.30, 126.70, 126.08, 126.00, 125.73, 125.66, 125.36, 123.10, 111.424, 36.02, 32.08, 31.74, 29.85, 29.82, 29.79, 29.71, 29.57, 29.52, 22.85, 14.28; HRMS (ESI) *m/z* calculated for $C_{45}H_{63}NNa$ $[M + Na]^+$ 640.4858, found 640.4840.

b. 4,4'-(1,5-dichloroanthracene-9,10-diyl)bis(2-(3,5-didodecylphenyl)naphtho[2,3-h]quinoline) (3). This intermediate was typically prepared according to modified versions of established procedures.[1,3] First, a mixture of previously-reported 1,5-dichloro-9,10-diethynylanthracene (**1**) (1.08 g, 3.66 mmol),[1] chloranil (1.99 g, 8.09 mmol), synthesized precursor (**2**) (6.80 g, 11.0 mmol), and boron trifluoride diethyl etherate ($BF_3{\bullet}OEt_2$) (1.34 mL, 10.9 mmol) in chloroform (184 mL) was combined in a reaction vessel and stirred under argon at 70 ºC for 48 hours. Next, the reaction mixture was cooled to room temperature and sequentially washed with a saturated aqueous sodium bicarbonate solution (200 mL × 3) and water (200 mL × 3). Subsequently, the organics were poured through a cotton plug, and the solvent was removed *in vacuo*. In turn, the obtained solids were dissolved in chloroform and precipitated from methanol. Last, the material was further purified by flash chromatography (100/0 to 70/30 hexanes/chloroform) using silica gel and isolated as two atropisomers, which were triturated with hot ethanol to yield pale yellow solids (2.43 g (**3**, *isomer 1* + *isomer 2*), 43% yield): (**3**, *isomer 1*): ^{1}H NMR (500 MHz, $CDCl_3$) δ 10.22 (s, 2H), 8.41 – 8.39 (m, 4H), 8.18 (s, 2H), 8.12 – 8.10 (m, 6H), 7.72 (d, *J* = 9.2 Hz, 2H), 7.68 – 7.59 (m, 6H), 7.53 (d, *J* = 7.0 Hz, 2H), 7.20 – 7.16 (m, 4H), 7.12 (d, *J* = 9.2 Hz, 2H), 2.81 (t, *J* = 7.9 Hz, 8H), 1.79 (quint, *J* = 7.6 Hz, 8H), 1.50 – 1.28 (m, 74H), 0.89 (t, *J* = 6.8 Hz, 12H); (**3,** *isomer 2*): ^{1}H NMR (500 MHz, $CDCl_3$) δ 10.21 (s, 2H), 8.43 (s, 2H), 8.40 – 8.38 (m, 2H), 8.13 – 8.11 (m, 2H), 8.07 (br s, 6H), 7.77 (d, *J* = 9.2 Hz, 2H), 7.67 – 7.62 (m, 4H), 7.58 (d, *J* = 9.1 Hz, 2H), 7.52 (d, *J* = 7.0 Hz, 2H), 7.22 (d, *J* = 9.2 Hz, 2H), 7.19 – 7.15 (m, 4H), 2.77 (t, *J* = 7.9 Hz, 8H), 1.76 (quint, *J* = 7.5 Hz, 8H), 1.47 – 1.26 (m, 75H), 0.87 (t,

J = 6.9 Hz, 12H); (**3,** *isomer 1*): ^{13}C NMR (126 MHz, $CDCl_3$) δ 155.28, 148.27, 147.16, 143.78, 139.73, 134.45, 133.44, 133.24, 132.49, 131.96, 131.44, 130.51, 130.41, 130.01, 129.49, 128.40, 128.06, 127.78, 127.42, 126.59, 126.55, 126.03, 125.29, 124.97, 123.34, 122.77, 36.42, 32.09, 31.95, 29.90, 29.88, 29.86, 29.83, 29.80, 29.72, 29.53, 22.85, 14.28; (**3**, *isomer 2*): ^{13}C NMR (126 MHz, $CDCl_3$) δ 155.40, 148.34, 147.14, 143.76, 139.72, 134.43, 133.47, 133.26, 132.50, 131.98, 131.48, 130.50, 130.41, 129.99, 129.49, 128.47, 128.08, 127.70, 127.47, 126.64, 126.59, 126.09, 126.03, 125.27, 124.97, 123.55, 122.51, 36.39, 32.08, 31.90, 29.88, 29.87, 29.84, 29.82, 29.78, 29.72, 29.68, 29.52, 29.42, 22.84, 14.27; MALDI *m/z* calculated for $C_{108}H_{131}Cl_2N_2$ $[M+H]^+$ 1525.97, found 1525.19.

c. 2,15-bis(3,5-didodecylphenyl)dibenzo[lm:e$_1$f$_1$]dipyrido[2,3,4-hi:2,3,4-a$_1$b$_1$]nonacene (4). This product was typically prepared according to modified versions of established procedures.[1] First, a mixture of the synthesized intermediate (**3**) (0.500 g, 0.33 mmol), powdered KOH (2.21 g, 39.3 mmol), and anhydrous quinoline (30 mL) was combined in a reaction vessel, thoroughly degassed for 15 minutes, and stirred under argon at 150 °C for 2 hours. Next, the reaction mixture was cooled to room temperature and poured into chloroform (50 mL). In turn, the resulting mixture was sequentially washed with water (50 mL), an aqueous solution of HCl (2.4 M, 50 mL × 3), water (50 mL × 3), and a saturated solution of aqueous sodium bicarbonate (50 mL). Subsequently, the organics were poured through a cotton plug, and the solvent was removed *in vacuo*. Then, the obtained solids were rinsed with methanol. Last, the material was purified by flash chromatography using deactivated silica gel (100/0 to 90/10 hexanes/ethyl acetate) and isolated as a dark blue/green solid (0.068 g, 14% yield): ^{1}H NMR (500 MHz, CS_2) δ 9.68 (s, 2H), 8.55 (d, J = 8.5 Hz, 2H), 8.47 (s, 2H), 8.40 (s, 2H), 8.27 (d, J = 7.4 Hz, 2H), 8.25 (s, 2H), 8.09 – 8.07 (m, 2H), 7.92 – 7.90 (m, 2H), 7.83 (s, 4H), 7.54 (t, J = 7.9 Hz, 2H), 7.47 – 7.43 (m, 4H), 7.02 (s, 2H), 2.72

(t, J = 7.7 Hz, 8H), 1.72 (quint, J = 7.5 Hz, 8H), 1.44 – 1.19 (m, 79H), 0.82 (t, J = 6.9 Hz, 12H); ^{13}C NMR (126 MHz, CS_2) δ 155.13, 147.55, 142.50, 139.31, 137.56, 132.97, 132.00, 131.52, 129.71, 129.27, 129.15, 129.09, 128.98, 128.62, 127.69, 127.58, 127.11, 126.94, 126.70, 126.16, 125.77, 125.68, 124.83, 124.75, 121.28, 121.00, 120.71, 118.09, 36.33, 32.07, 31.70, 29.95, 29.93, 29.89, 29.87, 29.72, 29.57, 23.05, 14.36; MALDI m/z calculated for $C_{108}H_{128}N_2$ $[M]^+$ 1453.01, found 1452.78 (initially), found 1453.04 (after > 2 years of storage).

3. General Analytical Methods.

a. Nuclear Magnetic Resonance (NMR) Spectroscopy Characterization. The precursors, intermediates, and product (**4**) were characterized with 1D ^{1}H NMR spectroscopy, 1D ^{13}C NMR spectroscopy, and 2D ^{1}H-^{1}H correlation spectroscopy (COSY) in the University of California, Irvine Nuclear Magnetic Resonance Spectroscopy Facility. The ^{1}H NMR, ^{13}C NMR, and ^{1}H-^{1}H COSY spectra were obtained with either a Bruker DRX500 spectrometer that was outfitted with a CryoProbe (Bruker TCI 500 MHz, 5 mm diameter tubes) or an Avance 600 spectrometer that was outfitted with a CryoProbe (Bruker CBBFO 600 MHz, 5 mm diameter tubes). The NMR experiments were typically performed at compound concentrations of ~ 1 mg/mL to ~ 10 mg/mL. The concentration-dependent chemical shifts were reported in ppm for the NMR spectra and were referenced as follows: for samples in $CDCl_3$, the ^{1}H NMR spectra were referenced to tetramethylsilane (TMS) at 0.00 or the residual $CHCl_3$ peak at 7.26 ppm, and the ^{13}C NMR spectra were typically referenced to the residual $CHCl_3$ peak at 77.16 ppm; for samples in CS_2, the ^{1}H NMR spectra were typically referenced to TMS at 0.00, and the ^{13}C NMR spectra were referenced to TMS at 0.00. Both the ^{1}H and ^{13}C NMR data were labeled with the chemical shift, multiplicity (s = singlet, d = doublet, t = triplet, q = quartet, quint = quintet, m = multiplet, and br s = broad

singlet), coupling constants in Hertz, and integration values. The NMR spectra were processed and analyzed by using the MestreNova software suite.

b. Mass Spectrometry Characterization. The precursors, intermediates, and product (**4**) were characterized with electrospray ionization (ESI) high-resolution mass spectrometry (HRMS) and/or matrix-assisted laser desorption/ionization-time of flight (MALDI-TOF) mass spectrometry in the University of California, Irvine Mass Spectrometry Facility. The HRMS measurements were typically performed on a Waters LCT Premier TOF spectrometer, and the MALDI-TOF measurements were typically performed on an AB SCIEX TOF/TOF™ 5800 mass spectrometer using a 349 nm Nd:YAG laser, with either TCNQ or dithranol as the matrix. The mass spectra were processed and analyzed by using the standard MassLynx and TOF/TOF Series Explorer software packages.

c. Chromatographic Characterization. Product (**4**) was characterized via size exclusion chromatography (SEC) with a refractive index detector. The measurements were performed with an Agilent Technologies 1260 Infinity Series separations module outfitted with two Agilent ResiPore columns connected in series (7.5 mm × 300 mm, 3 µm particle size). The separations module was connected to Agilent 1260 infinity dual angle light scattering, refractive index, and viscosity detectors in series. The typical conditions were: THF as the solvent, a temperature of 40 ºC, a flow rate of ~ 1.0 mL, an injection volume of ~ 25 – 100 µL, and a compound concentration of ~ 1.0 – ~ 2.5 mg/mL. The chromatography data was processed and analyzed by using the standard Agilent GPC/SEC Software packages.

B. Computational Analysis of the Nonacene-Like Molecule.

1. Density Functional Theory (DFT) Calculations. The restricted or conventional DFT and unrestricted broken symmetry DFT calculations were performed for the unprotonated

tetrabenzononacene (TBN, **4**) and the protonated tetrabenzononacene (PTBN, **4+2H⁺**) by using the Gaussian 16 (Revision B.01) software package.[5] First, the ground-state geometries, electronic structures, and molecular orbitals were calculated for TBN and PTBN with the restricted B3LYP functional and the 6-311G (d,p) basis set, which has been shown to furnish accurate predictions for comparable acenes and N-heteroacenes (note that the side chains were excluded for computational tractability).[1,6-13] The accuracy of the obtained geometries was verified by ensuring the absence of imaginary frequencies in the normal mode calculations. Second, the energies and molecular orbitals were recalculated for TBN and PTBN with the unrestricted broken symmetry B3LYP functional and the same basis set.[8,14] The obtained ground-state energies were within 10^{-6} eV of those calculated with the restricted B3LYP functional, with no mixing of different electron spin states (i.e., spin contamination), and all of the occupied molecular orbitals were doubly occupied, indicating a closed shell character for the unprotonated and protonated forms of the molecule. These combined calculations yielded the ground-state geometries and electronic structures for TBN and PTBN.

2. Time-Dependent Density Functional Theory (TDDFT) Calculations. The TDDFT calculations were performed for TBN, **4** and PTBN, **4+2H⁺** by using the Gaussian 16 (Revision B.01) software package.[5] Initially, the ground-state geometries and normal vibrational modes were obtained for TBN and PTBN from the restricted or conventional DFT calculations. By assuming that the excited-state and ground-state vibrational modes were identical, the vibronic spectra were calculated for TBN and PTBN by using the excited-state energy gradient with the vertical gradient (VG) Franck-Condon (FC) method, which has been shown to furnish accurate predictions for analogous pi-conjugated systems at reasonable computational cost.[15-18] The FC-TDDFT calculations for TBN and PTBN considered the excitations from the lowest-energy vibrational

states of the molecules' electronic ground states to the five electronic excited states within the experimentally-accessible energy range, so the predicted absorption spectra were obtained by averaging the vibronic spectra calculated for these five electronic excited states. The combined calculations yielded the excited-state electronic structures and optical properties of TBN and PTBN.

Additional calculations were performed for TBN and PTBN in order to evaluate the nature of their electronic transitions. For this purpose, the electronic transition density matrix $(p^{g\alpha})_{nm}$ between the ground state and the excited state was calculated for TBN and PTBN according to the following standard equation:

$$(p^{g\alpha})_{nm} = \langle \Psi_\alpha | c_m^\dagger c_n | \Psi_g \rangle \quad (1)$$

where Ψ_g is the wavefunction of the ground state, Ψ_α is the wavefunction of the excited state, $c_m^\dagger$ is the creation operator, c_n is the annihilation operator, *n* and *m* are the indices of the different atomic orbital basis functions.[19,20] Here, the application of a unitary transformation resulted in the construction of two new orbitals and simplified the qualitative description of the electronic transitions (i.e., for every hole in the occupied space, there was one corresponding particle in the virtual space).[19] This approach yielded the natural transition orbitals associated with the lowest energy excited state transitions for TBN and PTBN, which primarily feature highest occupied molecular orbital (HOMO) and lowest unoccupied molecular orbital (LUMO) character with weights of ~ 0.97. The calculations provided further insight into the excited state electronic structures and optical properties of TBN and PTBN.

C. Solution-Phase Spectroscopy of the Nonacene-Like Molecule.

1. Preparation of the Solutions. The TBN and PTBN solutions were prepared according to routine protocols.[1-3] In a general procedure, TBN solutions were prepared by dissolving TBN in

HPLC-grade chloroform (Thermo Fisher Scientific) at a typical concentration of ~ 40 μM. The PTBN solutions were prepared by diluting TBN in HPLC-grade chloroform with concentrated trifluoroacetic acid (TFA) (Thermo Fisher Scientific) (to a typical PTBN concentration of ~ 40 μM and a typical acid concentration of ~ 1.0 M). Such solutions were then used immediately or further diluted to variable concentrations of > ~ 5 μM. To titrate TBN, the described general procedure was followed to prepare solutions with a consistent ~ 30 μM TBN concentration but variable 0 M, 0.00001 M, 0.0001 M, 0.001 M, 0.01 M, 0.1 M, 0.2 M, 1 M, and 2 M TFA concentrations. To convert PTBN back to TBN, the described general procedure was followed to prepare a chloroform solution with a ~ 30 μM TBN concentration, a 1 M TFA concentration, and a 2 M sodium hydroxide (NaOH) (Thermo Fisher Scientific) concentration. In an analogous general procedure, TBN solutions were prepared by dissolving TBN in a 1:4 (v/v) mixture of Hi Sol 15 Aromatic 150 (Univar Solutions) and VM&P Naphtha 66 solvents (Gallade Chemical) at a typical concentration of ~ 40 μM. The PTBN solutions were prepared by diluting TBN in a 1:4 (v/v) mixture of Hi Sol 15 Aromatic 150 and VM&P Naphtha 66 with the acidic NEXAR™ sulfonated pentablock copolymer (NEXAR, Kraton Polymers) in the same solvent mixture (to a typical PTBN concentration of ~ 10 μM and a typical polymer weight percentage of ~ 2%). Such solutions were then used as prepared or further diluted to variable concentrations of > ~ 5 μM. When necessary, the 6,13-bis((trimethylsilyl)ethynyl)pentacene (TMS-pentacene) solutions were prepared by following the same general procedures. The various solutions were used for spectroscopic characterization and stability evaluation.

2. Ultraviolet-Visible-Near-Infrared (UV-Vis-NIR) Spectroscopy Characterization. The TBN and PTBN solutions were characterized with UV-Vis-NIR spectroscopy in the University of California, Irvine Laser Spectroscopy Laboratory. The measurements were performed with either

a Jasco V-670 UV-Vis-NIR Spectrophotometer, an Agilent Cary 60 UV-Vis Spectrophotometer, or a Shimadzu UV-1700 UV-Vis Spectrophotometer. All of the measurements were performed in ambient atmosphere at room temperature. The UV-Vis-NIR spectroscopy experiments were repeated for at least three independently-prepared solutions in all instances. The spectra were processed and analyzed with the Jasco Spectra Manager Suite, Cary WinUV, and/or Igor Pro software packages.

3. Ambient Photostability Evaluation. The TBN, PTBN, and TMS-pentacene solutions were characterized with UV-Vis-NIR spectroscopy during illumination in the University of California, Irvine Laser Spectroscopy Laboratory. In a general procedure, a TBN, PTBN, or TMS-pentacene solution was transferred to a sealed cuvette (~ 1 cm path length). The solution was then directly illuminated with a high-intensity Xenon lamp (8W) light source outfitted with a neutral density filter (power of ~ 460 mW over a 1 cm^2 detector area). The UV-Vis-NIR absorption spectra were measured for the solution in an Agilent Cary 60 UV-Vis Spectrophotometer at different time intervals throughout illumination. For the TBN solution and the TMS-pentacene solution, the measurements were continued until complete degradation (i.e., a nearly complete loss of the peak absorbance), but for the PTBN solution, the measurements were typically continued for > 24 hours until partial degradation (i.e., partial loss of the peak absorbance). The measurements enabled calculation of the solution-phase half-life (defined as the time necessary to reduce the maximum peak absorbance intensity by 2-fold) for TBN, PTBN, and TMS-pentacene under illumination. All of the measurements were performed in ambient atmosphere at room temperature. The described procedure was repeated for at least three independently-prepared TBN, PTBN, and TMS-pentacene solutions. The spectra were processed and analyzed with the Igor Pro software package.

4. Fluorescence Spectroscopy Characterization. The TBN and PTBN solutions were characterized with fluorescence spectroscopy in the University of California, Irvine Laser Spectroscopy Laboratory. The measurements were performed with a Cary Eclipse Fluorescence Spectrophotometer. For the TBN solutions, the excitation wavelengths were 365 nm and 667 nm, and for the PTBN solutions, the excitation wavelengths were 365 nm and 842 nm. The excitation and emission slit widths were typically 5 nm. All of the measurements were performed in ambient atmosphere at room temperature. The fluorescence spectroscopy experiments were repeated for at least three independently-prepared solutions in all instances. The spectra were processed and analyzed with the Cary Eclipse and Igor Pro software packages.

D. Fabrication, Mechanical Actuation, and Characterization of the Tri-Layer Architectures from the Nonacene-Like Molecule.

1. Fabrication of the Tri-Layer Architectures. The tri-layer architectures with TBN- or PTBN-based central layers were fabricated on a benchtop according to modified versions of literature procedures, as illustrated in Supplementary Figure 19.[21,22] First, an acrylic elastomer membrane (VHB 4905, 3M) was mounted on a size-adjustable holder and equiaxially stretched by ~ 1,600 % with respect to its initial area. Next, TBN in a 1:4 (v/v) mixture of Hi Sol 15 Aromatic 150 and VM&P Naphtha 66 solvents (with a typical TBN concentration of ~ 700 μM and without any additives) was dropcast onto the pre-stretched membrane to form a square-shaped, bright blue film (size of ~ 3.5 cm x ~ 3.5 cm), and the solvent was allowed to completely evaporate. Alternatively, PTBN/NEXAR in a 1:4 (v/v) mixture of Hi Sol 15 Aromatic 150 and VM&P Naphtha 66 solvents (with a typical PTBN concentration of ~ 350 μM and with the acidic NEXAR™ sulfonated pentablock copolymer additive to ensure protonation of the molecule) was dropcast onto the pre-stretched membrane to form a square-shaped, dark-brown film (length of ~

3.5 cm), and the solvent was allowed to completely evaporate. In tandem, a ~ 5 weight % NEXAR^{TM} sulfonated pentablock copolymer solution was spincast onto a silicon substrate, heat-treated on the substrate at a temperature of 60 °C, and then delaminated from the substrate as a transparent film. Subsequently, the square NEXAR^{TM} sulfonated pentablock copolymer film (length of ~ 3.5 cm) was laminated directly on top of either 1) a square-shaped, bright blue TBN film or 2) a square-shaped, dark brown PTBN/NEXAR film. Last, the tri-layer architectures were mechanically contracted by ~ 40 % with respect to the initial lengths of the square-shaped TBN or PTBN/NEXAR layers. The resulting completed architectures were used for the mechanical actuation experiments.

2. Mechanical Actuation of the Tri-Layer Architectures. The tri-layer architectures with TBN- or PTBN-based central layers were mechanically actuated in a size-adjustable holder, as previously reported.[21,22] The holder enabled the application and release of equiaxial strain. The lengths of the architectures' square-shaped blue or brown regions were increased by ~ 67 % for their actuated states and were then contracted by ~ 40 % for their unactuated (i.e., initial) states. All of the experiments were performed under standard indoor lighting in ambient atmosphere at room temperature. The unactuated or actuated TBN architectures and unactuated or actuated PTBN/NEXAR architectures were used for the subsequent characterization experiments.

3. Digital Camera Characterization of the Tri-Layer Architectures. The visible appearances of the unactuated and actuated tri-layer architectures with TBN- or PTBN-based central layers were characterized via digital camera imaging. The images were obtained with either a built-in iPhone Digital Camera or with a Canon PowerShot SX520 HS Digital Camera. All of the experiments were performed under standard indoor lighting in ambient atmosphere at room temperature. The imaging experiments were repeated for at least three independent unactuated or

actuated TBN architectures and at least three independent unactuated or actuated PTBN/NEXAR architectures. The images were processed and analyzed with the Adobe Photoshop software package.

4. Morphological Characterization of the Tri-Layer Architectures. The topographies of unactuated and actuated tri-layer architectures with TBN- or PTBN-based central layers were characterized with atomic force microscopy (AFM). The images were obtained with an Asylum Cypher ES Atomic Force Microscope (AFM) operating in tapping mode. All of the measurements were performed in ambient atmosphere at room temperature. The morphological characterization experiments were repeated for at least three independent unactuated or actuated TBN architectures and at least three independent unactuated or actuated PTBN/NEXAR architectures. The images were processed and analyzed with the Gwyddion software package.

5. Spectroscopic Characterization of the Tri-Layer Architectures. The spectroscopic properties of unactuated and actuated tri-layer architectures with TBN- or PTBN-based central layers were characterized with UV-Vis-NIR spectroscopy. The measurements were performed with a Jasco V-670 UV-Vis-NIR Spectrophotometer outfitted with a Jasco ILN-925 150 mm integrating sphere. This spectrophotometer featured a rectangular-shaped port with a length of ~ 0.9 cm and a width of ~ 1.3 cm in transmission mode and a square-shaped port with a length of ~ 1.6 cm length in reflection mode, and the unactuated and actuated tri-layer architectures' square-shaped blue or brown regions were large enough to completely cover the ports in the transmission and reflection modes. The measurements were performed at normal incidence in transmission mode or at an incidence angle of 5° in reflection mode. The measurements were referenced to Jasco Spectralon standards, as appropriate. The absorptance was calculated from the transmittance and reflectance measurements according to the following standard equation:

$$T\ (\%) + R\ (\%) + A\ (\%) = 100\ \% \quad (2)$$

where T is the total transmittance, R is the total reflectance, and A is the total absorptance.[21-23] All of the measurements were performed under standard indoor lighting in ambient atmosphere at room temperature. The UV-Vis-NIR spectroscopy experiments were repeated for at least three independent unactuated or actuated TBN architectures and at least three independent unactuated or actuated PTBN/NEXAR architectures. The spectra were processed and analyzed with the Jasco Spectra Manager™, Igor Pro, and Python software packages.

E. Fabrication, Electrical Actuation, and Characterization of the Quad-layer Devices from the Nonacene-Like Molecule.

1. Fabrication of the Quad-Layer Devices. The TBN- and PTBN-based quad-layer devices were fabricated on a benchtop according to modified versions of literature procedures, as illustrated in Supplementary Figure 21.[21,22] First, an acrylic elastomer membrane (VHB 4905, 3M) was mounted on a size-adjustable holder and equiaxially stretched by ~ 1,600 % with respect to its initial area. Next, PTBN/NEXAR in a 1:4 (v/v) mixture of Hi Sol 15 Aromatic 150 and VM&P Naphtha 66 solvents was dropcast onto the pre-stretched membrane to form a circular, dark-brown active region (typical diameters of ~ 0.5 cm), with the solvent allowed to completely evaporate. Additionally, TBN in a 1:4 (v/v) mixture of Hi Sol 15 Aromatic 150 and VM&P Naphtha 66 solvents was dropcast onto the pre-stretched membrane to form an annular, dark-blue active region (typical inner diameters of ~ 0.5 cm and typical outer diameters of ~ 1.1 cm), with the solvent allowed to completely evaporate. In tandem, NEXAR™ sulfonated pentablock copolymer solutions were spincast onto silicon substrates, heat-treated on the substrates at a temperature of 60 °C, and then delaminated from the substrates as transparent proton-conducting electrodes. Subsequently, two identical circular proton-conducting polymer electrodes (typical diameters of ~ 1.2 cm) featuring rectangular extensions were laminated directly on top of the blue-brown

annulated circle region and on the bottom of the elastomer membrane. In turn, the quad-layer structure was mechanically contracted by ~ 40 % with respect to the initial diameters of the two circular electrodes. Last, two identical rectangular aluminum foil electrical leads were connected to both the top and bottom proton-conducting polymer electrodes. The resulting completed devices were used for the electrical actuation experiments.

2. Electrical Actuation of the Devices. The TBN- and PTBN-based quad-layer devices were electrically actuated in a size-adjustable holder, as previously reported.[21,22] The actuation was performed with a home-built high-voltage power supply consisting of a Stanford Research DS345 function generator, Texas Instruments OPA 548 an operational amplifier, and a EMCO E80 high-voltage converter. This voltage source enabled the application of variable-voltage and variable-frequency waveforms during monitoring of the devices with either a built-in iPhone Digital Camera or with a Canon PowerShot SX520 HS Digital Camera. The areal strain was calculated from the obtained movies and images according to the following equation:

$$\textit{Areal Strain (\%)} = (A_1 - A_0) / A_0 \times 100\ \% \quad (3)$$

where A_0 is the area of the active region before actuation and A_1 is the area of the active region after actuation. All of the measurements were performed under standard indoor lighting in ambient atmosphere at room temperature. The electrical actuation experiments were repeated for at least three (and as many as ten) independent devices in all instances. The obtained movies and images were processed and analyzed with the Apple QuickTime Player, MATLAB, and Adobe Photoshop software packages.

3. Visible Characterization of the Devices. The visible appearance of the TBN- and PTBN-based quad-layer devices was characterized both without and with actuation via digital camera imaging, as illustrated in Figure 4A. The movies and images were obtained with either a built-in iPhone Digital Camera or with a Canon PowerShot SX520 HS Digital Camera. The visible

lightness modulation for our devices' active regions (i.e., blue annuli or brown circles) were calculated from the obtained digital camera images according to the following equation:[24,25]

$$Lightness\ modulation\ (\%) = \frac{Image\ pixel\ values\ (final\ state) - Image\ pixel\ values\ (initial\ state)}{Image\ pixel\ values\ (initial\ state)} \qquad (4)$$

The mean pixel values required for these calculations were auto-calculated from the RGB channels of the images using the histogram function in the Adobe Photoshop software package. For the lightness modulation as a function of the applied voltage in Figure 4C, the initial state pixel values were extracted from images of the unactuated devices at 0 kV; for the lightness modulation as a function of time in Figure 4D, the initial state pixel values were extracted from images of the devices at 0 sec; and for the lightness modulation as a function of the cycle number in Figure 4E and Supplementary Table 3, the initial state pixel values were extracted from images of the devices at cycle number zero. The experiments were performed for devices positioned above a white printer paper background under standard indoor lighting in ambient atmosphere at room temperature. The imaging experiments were repeated for at least three (and as many as ten) independent devices. The obtained movies and images were processed and analyzed with the Apple QuickTime Player, MATLAB, and Adobe Photoshop software packages.

4. Near-Infrared Characterization of the Devices. The near-infrared contrast of the TBN- and PTBN-based quad-layer devices was characterized both without and with actuation via near-infrared camera imaging, as illustrated in Figure 5A. The movies and images were obtained with a Raspberry Pi NoIR Camera Module v2 outfitted with a Hoya RM-72 infrared filter. The near-infrared contrast changes for our devices' active regions (i.e., annuli or circles) were calculated from the obtained near-infrared camera images according to the following equation:

$$Contrast\ change\ (\%) = \frac{Image\ pixel\ values\ (final\ state) - Image\ pixel\ values\ (initial\ state)}{Image\ pixel\ values\ (initial\ state)} \qquad (5)$$

The mean pixel values required for these calculations were auto-calculated from the RGB channels of the images using the histogram function in the Adobe Photoshop software package. The initial and final state contrast values were extracted from images of the unactuated and actuated devices, respectively. The experiments were performed for devices positioned above a black benchtop background with illumination from a custom-built near-infrared Xtra LED array in ambient atmosphere at room temperature. The imaging experiments were repeated for at least three independent devices. The obtained movies and images were processed and analyzed with the Apple QuickTime Player, MATLAB, and Adobe Photoshop software packages.

5. Fluorescence Characterization of the Devices. The fluorescence signal intensity of the TBN- and PTBN-based quad-layer devices was characterized both without and with actuation via digital camera imaging, as illustrated in Figure 5C. The movies and images were obtained with a Canon PowerShot SX520 HS Digital Camera. The fluorescence signal intensity changes for our devices' active regions (i.e., annuli or circles) were calculated from the obtained digital camera images according to the following equation:

$$Signal\ intensity\ change\ (\%) = \frac{Image\ pixel\ values\ (final\ state) - Image\ pixel\ values\ (initial\ state)}{Image\ pixel\ values\ (initial\ state)} \tag{6}$$

The mean pixel values required for these calculations were auto-calculated from the RGB channels of the images using the histogram function in the Adobe Photoshop software package. The initial and final state intensity values were extracted from images of the unactuated and actuated devices, respectively. The experiments were performed for devices positioned above a white printer paper background with illumination from an Analytik Jena UVP EL Series ultraviolet lamp in ambient atmosphere at room temperature. The imaging experiments were repeated for at least three independent devices. The obtained movies and images were processed and analyzed with the Apple QuickTime Player, MATLAB, and Adobe Photoshop software packages.

II. Supplementary Figures

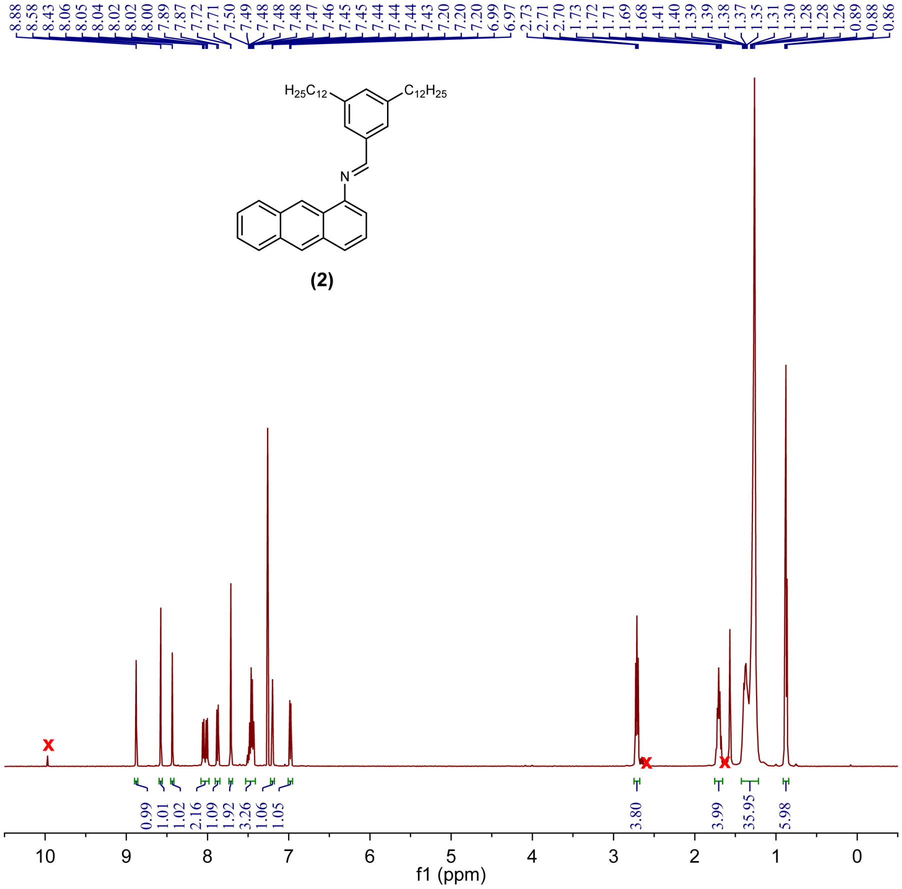

	Parameter	Value
1	Comment	1H spectrum
2	Origin	UXNMR, Bruker Analytische Messtechnik GmbH
3	Instrument	cryo500
4	Author	
5	Solvent	CDCl3
6	Temperature	298.0
7	Pulse Sequence	zg30
8	Experiment	1D
9	Probe	5 mm CPTCI 1H-13C/ 15N/ 2H Z-GRD Z44881/ 0020
10	Number of Scans	32
11	Receiver Gain	5.7
12	Relaxation Delay	0.1000
13	Pulse Width	7.5000
14	Presaturation Frequency	
15	Spectrometer Frequency	500.22
16	Spectral Width	8012.8
17	Lowest Frequency	-536.5
18	Nucleus	1H
19	Acquired Size	40864
20	Spectral Size	65536

Supplementary Figure 1. The ^{1}H NMR spectrum for (**2**). Note that the red "x" corresponds to residual starting materials.

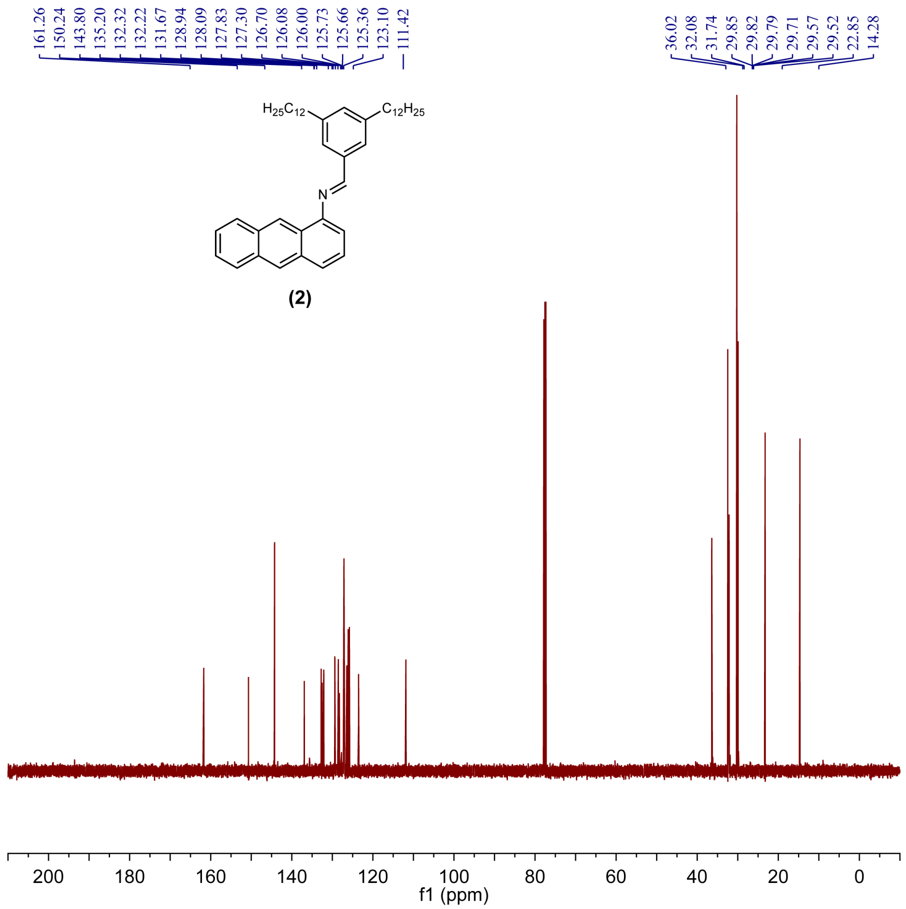

	Parameter	Value
1	Comment	Z-restored spin-echo 13C spectrum with 1H decoupling
2	Origin	UXNMR, Bruker Analytische Messtechnik GmbH
3	Instrument	cryo500
4	Author	
5	Solvent	CDCl3
6	Temperature	298.0
7	Pulse Sequence	SpinEchopg30gp.prd
8	Experiment	1D
9	Probe	5 mm CPTCI 1H-13C/ 15N/ 2H Z-GRD Z44881/ 0020
10	Number of Scans	608
11	Receiver Gain	7298.2
12	Relaxation Delay	0.2500
13	Pulse Width	16.5500
14	Presaturation Frequency	
15	Spectrometer Frequency	125.79
16	Spectral Width	30303.0
17	Lowest Frequency	-1315.7
18	Nucleus	13C
19	Acquired Size	32768
20	Spectral Size	65536

Supplementary Figure 2. The ^{13}C NMR spectrum for (**2**).

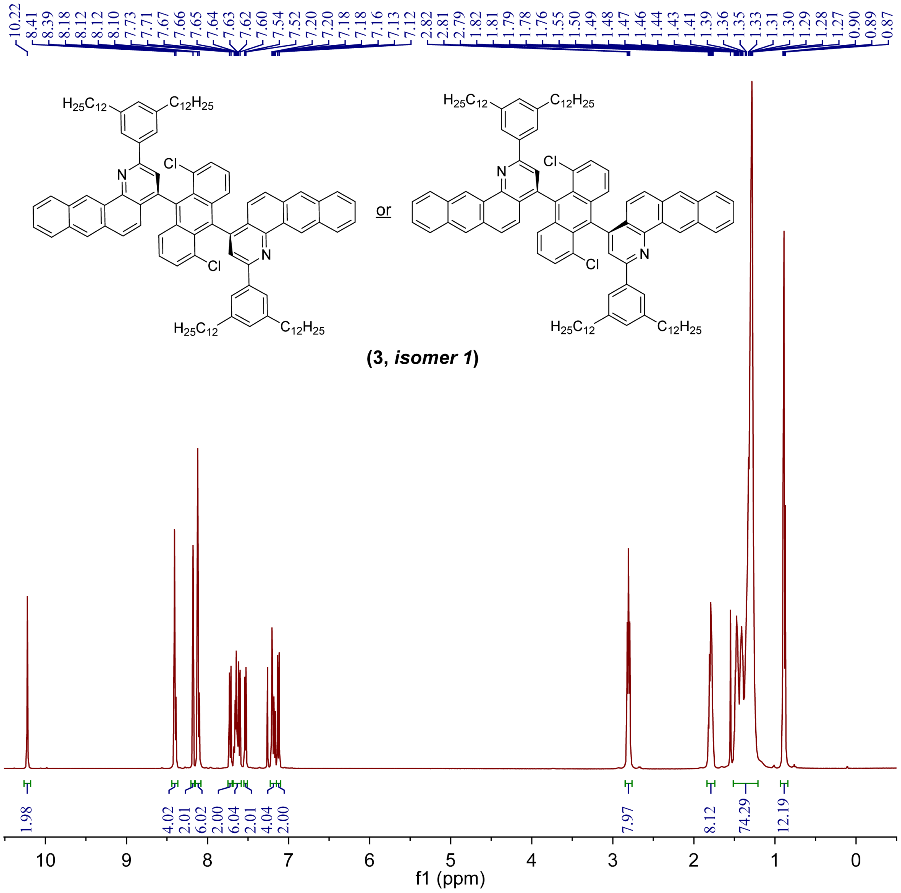

	Parameter	Value
1	Comment	1H spectrum
2	Origin	UXNMR, Bruker Analytische Messtechnik GmbH
3	Spectrometer	cryo500
4	Author	
5	Solvent	CDCl3
6	Temperature	298.0
7	Pulse Sequence	zg30
8	Experiment	1D
9	Probe	5 mm CPTCI 1H-13C/ 15N/ 2H Z-GRD Z44881/ 0020
10	Number of Scans	19
11	Receiver Gain	5.0
12	Relaxation Delay	0.1000
13	Pulse Width	7.5000
14	Presaturation Frequency	
15	Spectrometer Frequency	500.22
16	Spectral Width	8012.8
17	Lowest Frequency	-504.9
18	Nucleus	1H
19	Acquired Size	40864
20	Spectral Size	131072

Supplementary Figure 3. The ^{1}H NMR spectrum for (**3**, *isomer 1*).

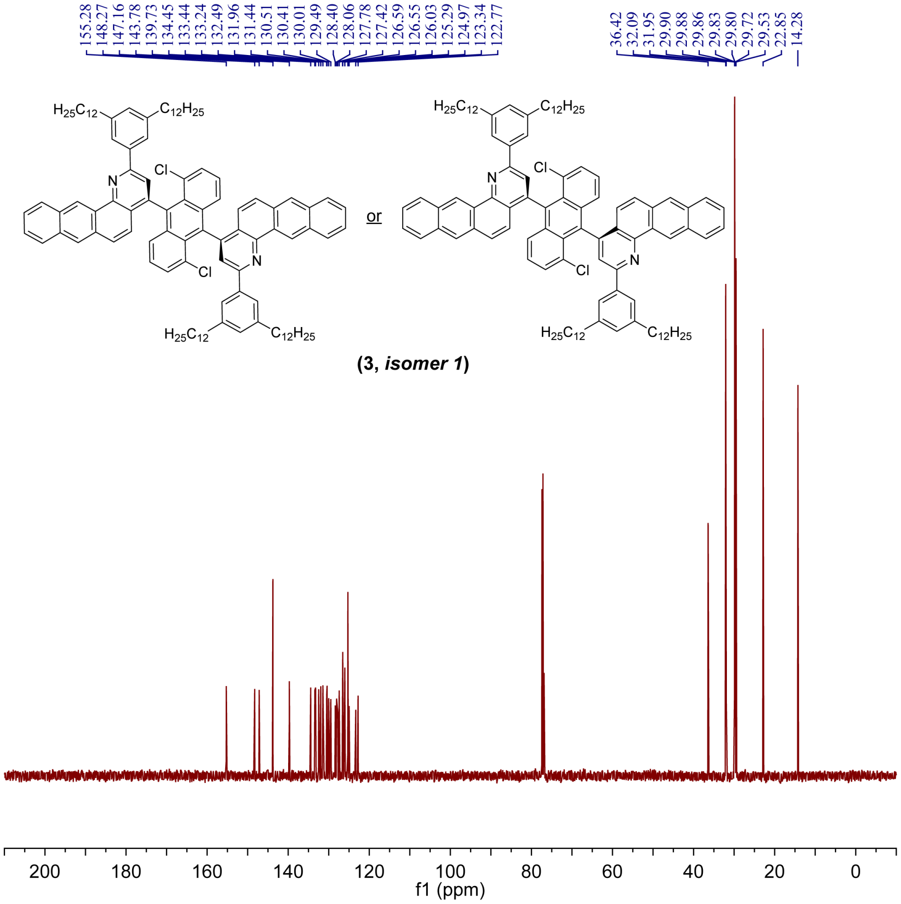

	Parameter	Value
1	Comment	Z-restored spin-echo 13C spectrum with 1H decoupling
2	Origin	UXNMR, Bruker Analytische Messtechnik GmbH
3	Spectrometer	cryo500
4	Author	
5	Solvent	CDCl3
6	Temperature	298.0
7	Pulse Sequence	SpinEchopg30gp.prd
8	Experiment	1D
9	Probe	5 mm CPTCI 1H-13C/ 15N/ 2H Z-GRD Z44881/ 0020
10	Number of Scans	220
11	Receiver Gain	7298.2
12	Relaxation Delay	0.2500
13	Pulse Width	16.5500
14	Presaturation Frequency	
15	Spectrometer Frequency	125.79
16	Spectral Width	30303.0
17	Lowest Frequency	-1315.7
18	Nucleus	13C
19	Acquired Size	32768
20	Spectral Size	65536

Supplementary Figure 4. The ^{13}C NMR spectrum for (**3**, *isomer 1*).

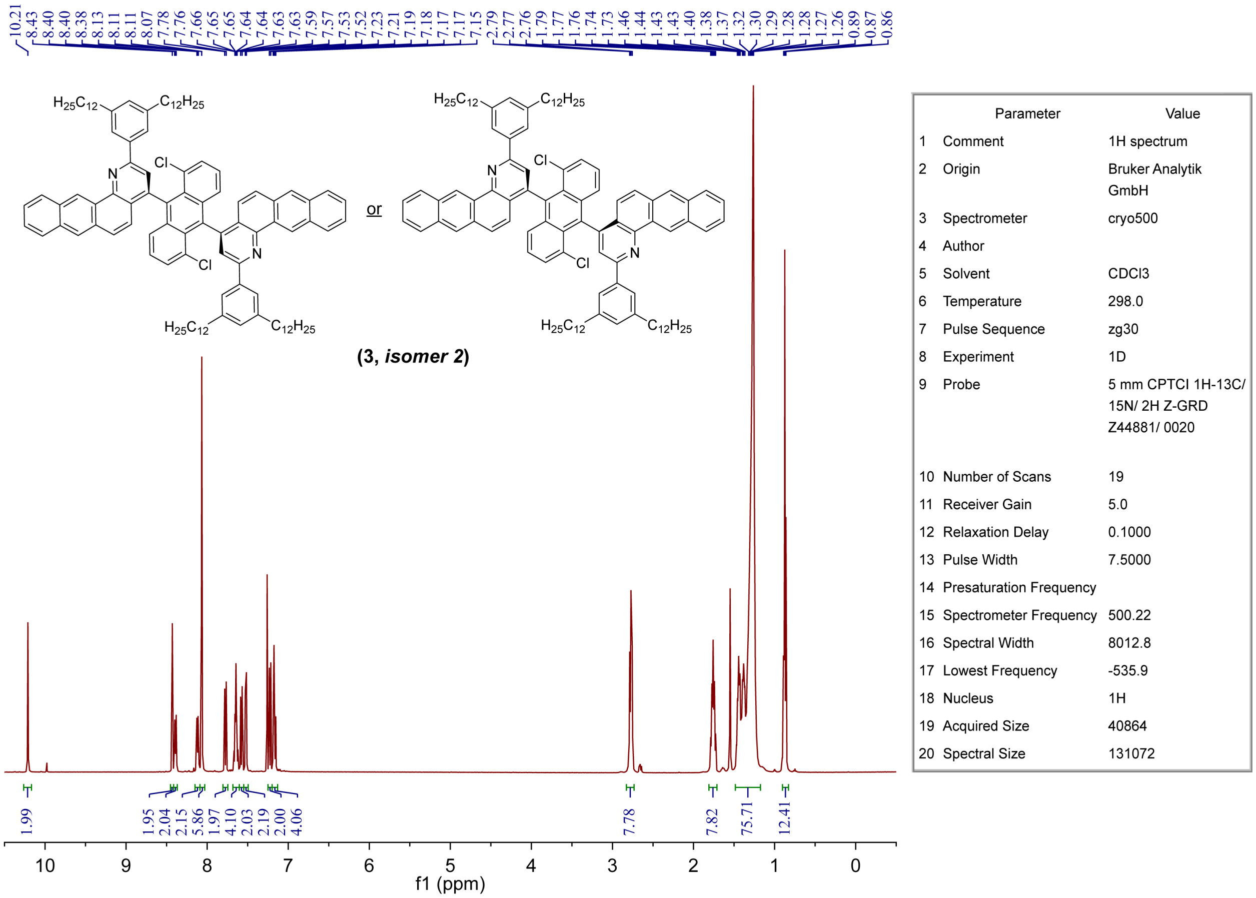

Supplementary Figure 5. The ^{1}H NMR spectrum for (**3**, *isomer 2*).

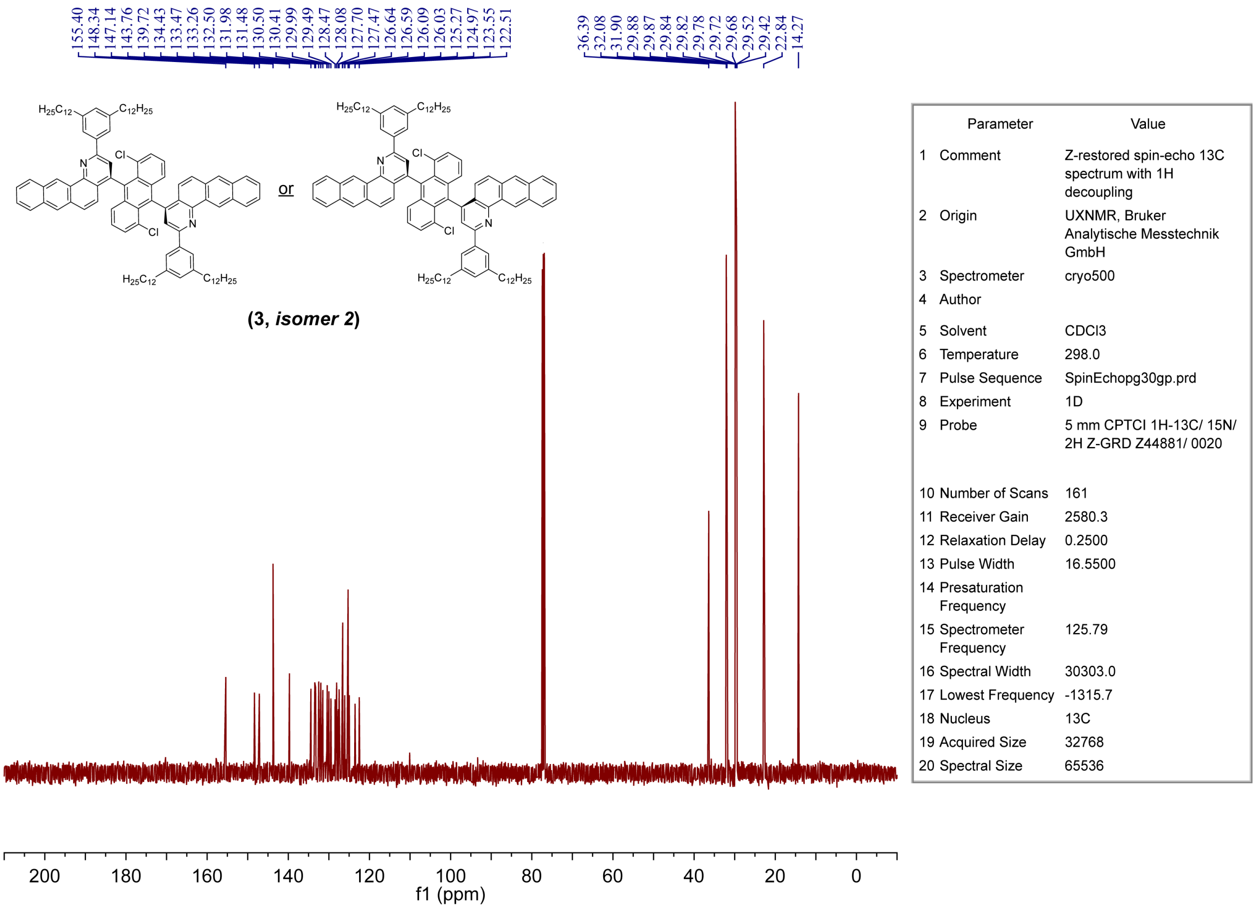

Supplementary Figure 6. The ^{13}C NMR spectrum for (**3**, *isomer 2*).

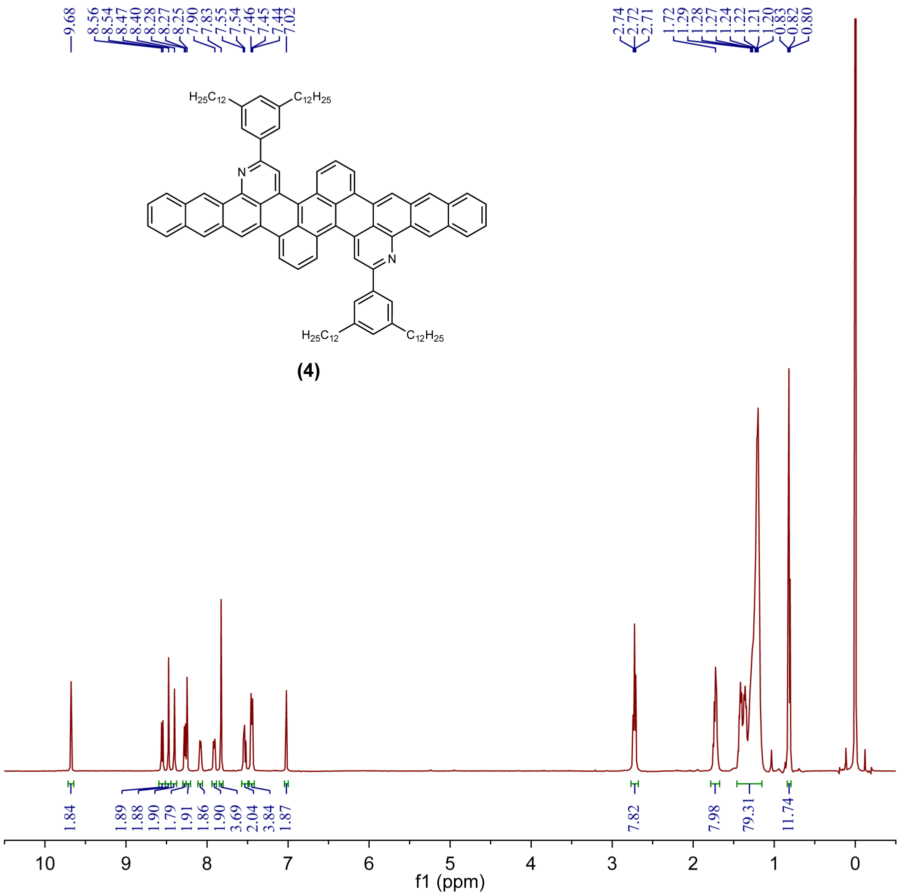

	Parameter	Value
1	Comment	1H spectrum
2	Origin	UXNMR, Bruker Analytische Messtechnik GmbH
3	Spectrometer	cryo500
4	Author	
5	Solvent	CS2
6	Temperature	298.0
7	Pulse Sequence	zg30
8	Experiment	1D
9	Probe	5 mm CPTCI 1H-13C/ 15N/ 2H Z-GRD Z44881/ 0020
10	Number of Scans	16
11	Receiver Gain	6.3
12	Relaxation Delay	0.1000
13	Pulse Width	7.5000
14	Presaturation Frequency	
15	Spectrometer Frequency	500.22
16	Spectral Width	8012.8
17	Lowest Frequency	-699.0
18	Nucleus	1H
19	Acquired Size	40864
20	Spectral Size	131072

Supplementary Figure 7. The ^{1}H NMR spectrum for (**4**).

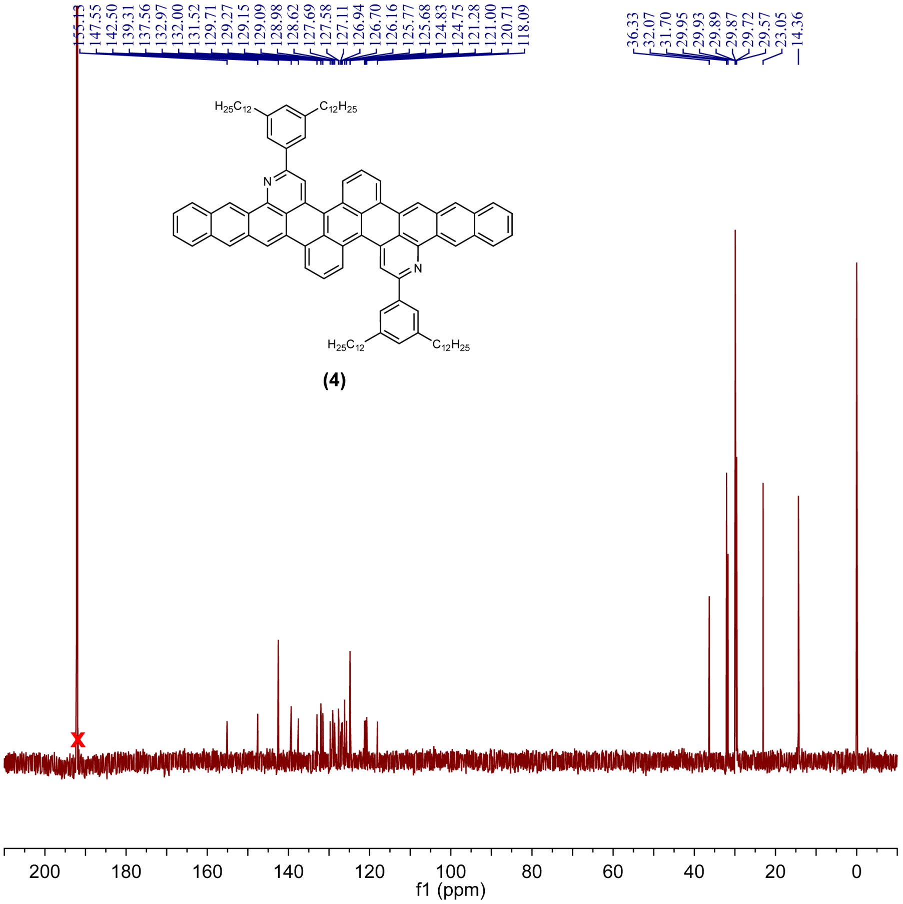

	Parameter	Value
1	Comment	Z-restored spin-echo 13C spectrum with 1H decoupling
2	Origin	UXNMR, Bruker Analytische Messtechnik GmbH
3	Spectrometer	cryo500
4	Author	
5	Solvent	CS2
6	Temperature	298.0
7	Pulse Sequence	SpinEchopg30gp.prd
8	Experiment	1D
9	Probe	5 mm CPTCI 1H-13C/ 15N/ 2H Z-GRD Z44881/ 0020
10	Number of Scans	400
11	Receiver Gain	3649.1
12	Relaxation Delay	1.0000
13	Pulse Width	16.5500
14	Presaturation Frequency	
15	Spectrometer Frequency	125.79
16	Spectral Width	30303.0
17	Lowest Frequency	-1410.3
18	Nucleus	13C
19	Acquired Size	32768
20	Spectral Size	65536

Supplementary Figure 8. The ^{13}C NMR spectrum for (**4**). Note that the red "x" corresponds to carbon disulfide.

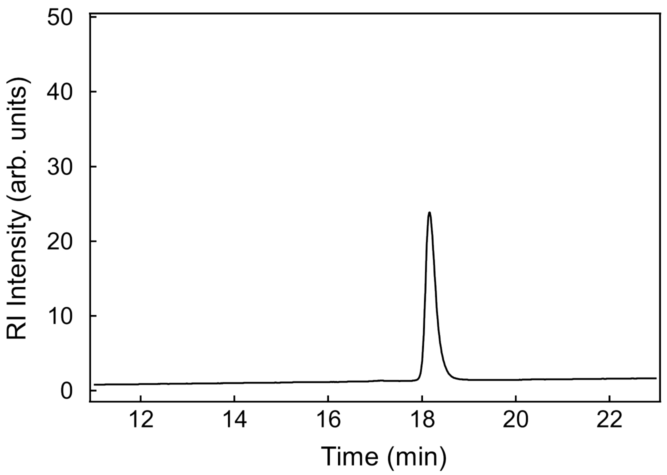

Supplementary Figure 9. The SEC-RI chromatogram for (**4**). The chromatogram was obtained in tetrahydrofuran.

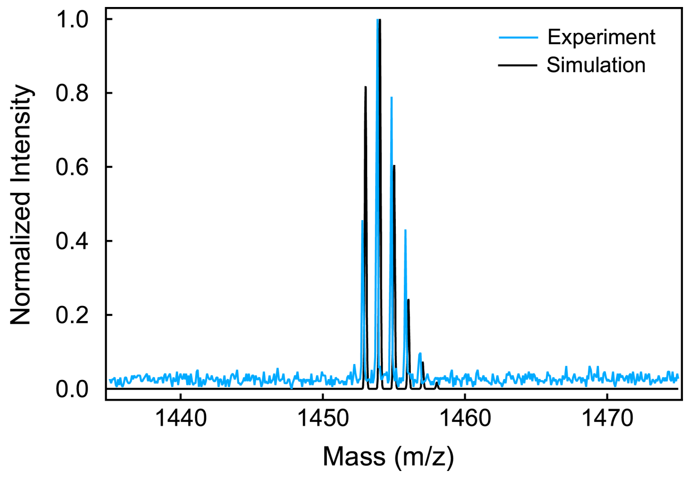

Supplementary Figure 10. The experimental (blue) and simulated (black) MALDI mass spectra for (**4**).

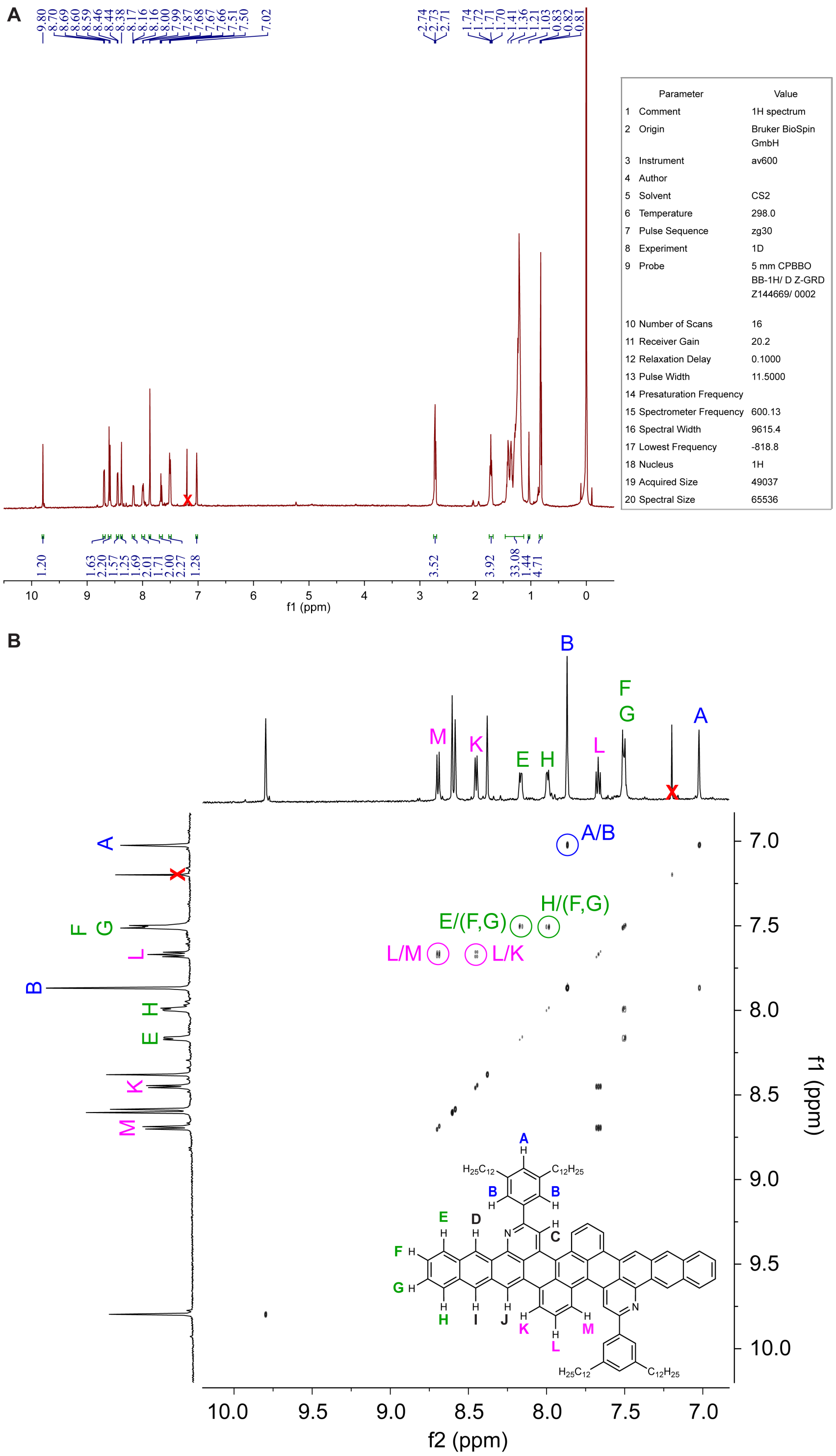

Supplementary Figure 11. (**A**) The 1D ^{1}H NMR spectrum for (**4**). (**B**) The 2D ^{1}H-^{1}H COSY spectrum for (**4**). The inset shows the molecular structure of (**4**) with the colored letter labels indicating specific protons. Note that the red "x" corresponds to residual chloroform.

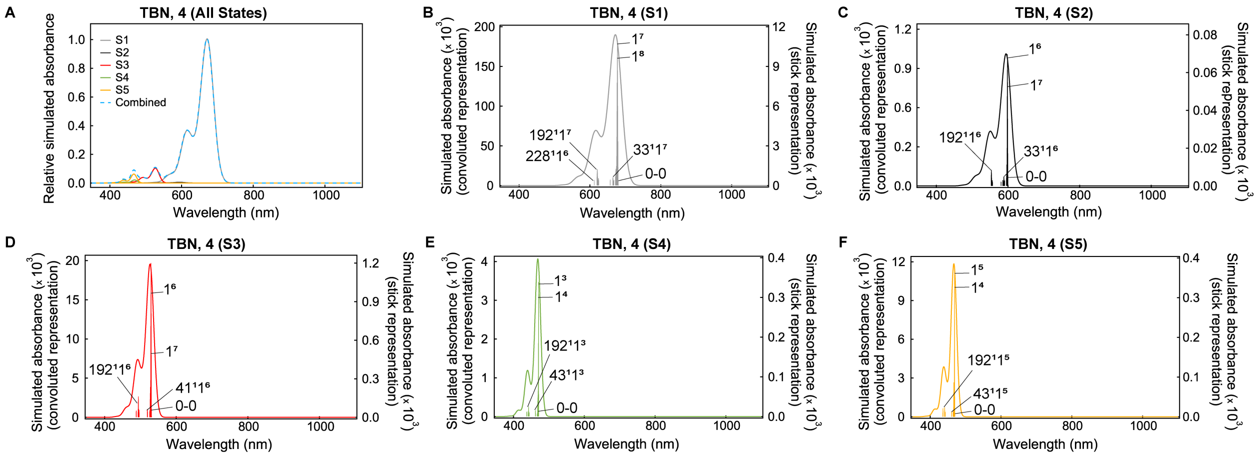

Supplementary Figure 12. (**A**) The theoretical UV-Vis-NIR absorption spectra (blue dashed line) calculated for the five electronic excited states S1 (gray solid line), S2 (black solid line), S3 (red solid line), S4 (green solid line), and S5 (orange solid line) of TBN. (**B**) The theoretical UV-Vis-NIR absorption spectrum calculated for excited state S1. (**C**) The theoretical UV-Vis-NIR absorption spectrum calculated for excited state S2. (**D**) The theoretical UV-Vis-NIR absorption spectrum calculated for excited state S3. (**E**) The theoretical UV-Vis-NIR absorption spectrum calculated for excited state S4. (**F**) The theoretical UV-Vis-NIR absorption spectrum calculated for excited state S5. The excited states S1, S2, S3, S4, and S5 are shown both as stick vibronic bands and as a convoluted envelope with applied Gaussian broadening. The vibronic progressions are labeled as "n^x", where n indicates the excited-state normal mode and x indicates the quanta deposited on the normal mode.

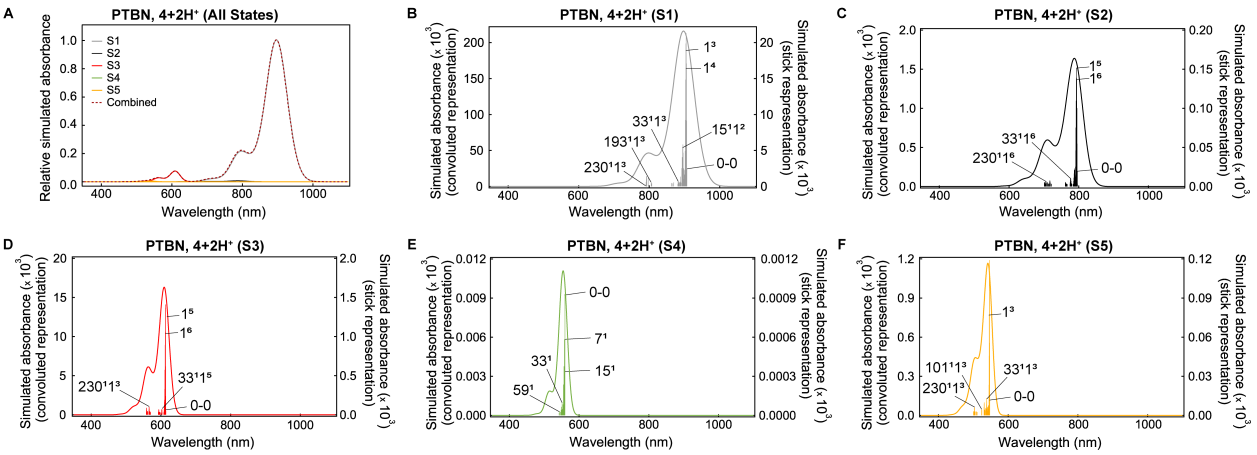

Supplementary Figure 13. (**A**) The theoretical UV-Vis-NIR absorption spectra (brown dashed line) calculated for the five electronic excited states S1 (gray solid line), S2 (black solid line), S3 (red solid line), S4 (green solid line), and S5 (orange solid line) of PTBN. (**B**) The theoretical UV-Vis-NIR absorption spectrum calculated for excited state S1. (**C**) The theoretical UV-Vis-NIR absorption spectrum calculated for excited state S2. (**D**) The theoretical UV-Vis-NIR absorption spectrum calculated for excited state S3. (**E**) The theoretical UV-Vis-NIR absorption spectrum calculated for excited state S4. (**F**) The theoretical UV-Vis-NIR absorption spectrum calculated for excited state S5. The excited states S1, S2, S3, S4, and S5 are shown both as stick vibronic bands and as a convoluted envelope with applied Gaussian broadening. The vibronic progressions are labeled as "n^x", where n indicates the excited-state normal mode and x indicates the quanta deposited on the normal mode.

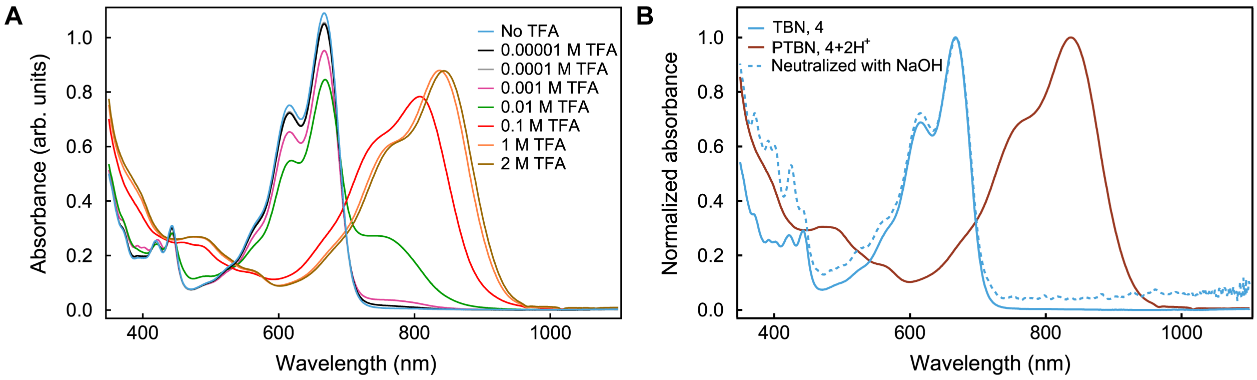

Supplementary Figure 14. (**A**) The experimental UV-Vis-NIR absorption spectra measured for TBN in chloroform at different TFA concentrations. (**B**) The experimental UV-Vis-NIR absorption spectra measured for TBN in chloroform without any added acid (blue solid line), PTBN in chloroform that was protonated with TFA (brown solid line), and TBN in chloroform that was protonated with TFA but then neutralized with NaOH (blue dashed line). Note that the absorption spectra shown in (B) were normalized to unity for clarity.

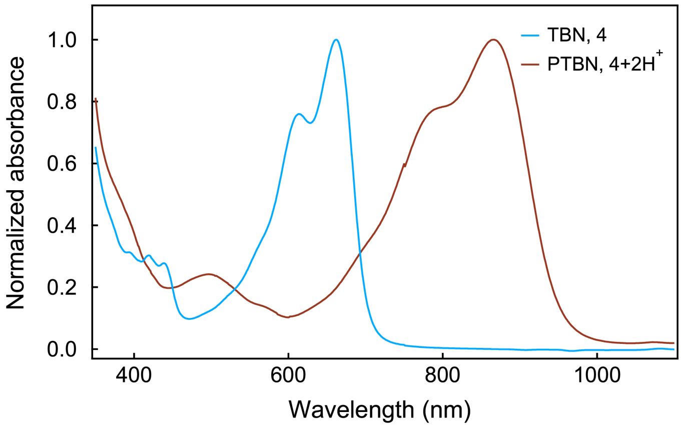

Supplementary Figure 15. The experimental UV-Vis-NIR absorption spectra measured for TBN without any added acid (blue line) and for PTBN that was protonated with the acidic NEXAR™ sulfonated pentablock copolymer (brown line) in a 1:4 (v/v) mixture of Hi Sol 15 Aromatic 150 and VM&P Naphtha 66 solvents. Note that the absorption spectra were normalized to unity for clarity.

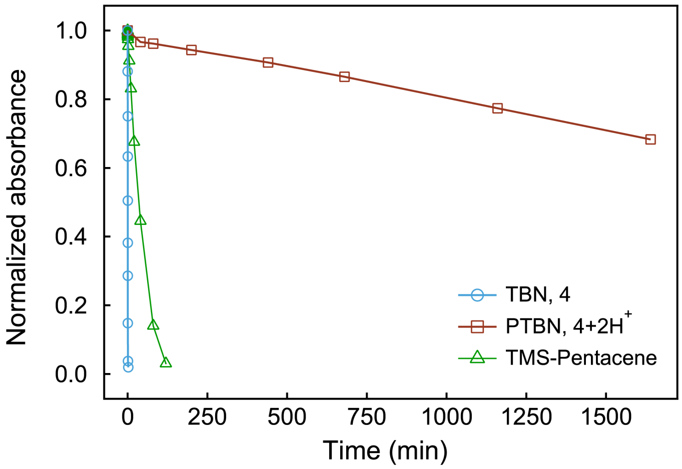

Supplementary Figure 16. A plot of the normalized peak absorbances as a function of time for TBN (blue circles), PTBN (brown squares), and TMS-pentacene (green triangles) in chloroform.

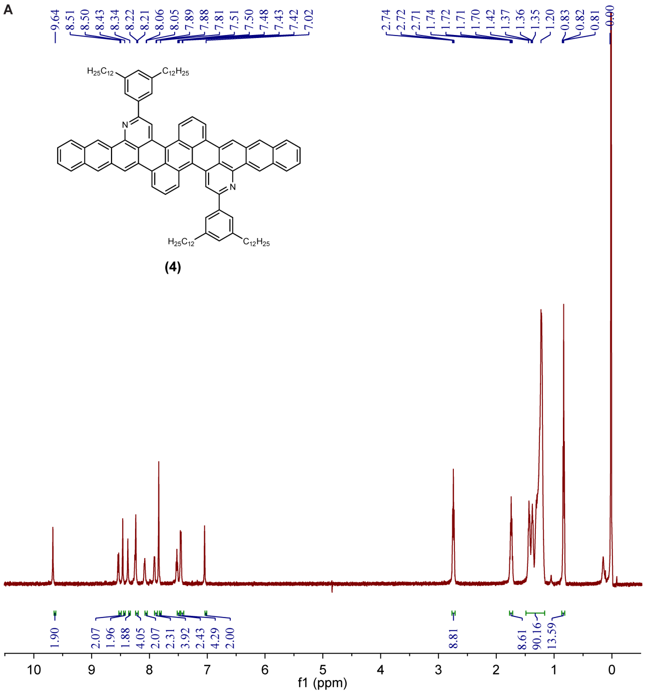

Parameter		Value
1	Comment	1H spectrum
2	Origin	Bruker BioSpin GmbH
3	Instrument	av600
4	Solvent	CS2
5	Temperature	298.0
6	Pulse Sequence	zg30
7	Experiment	1D
8	Probe	5 mm CPBBO BB-1H/ D Z-GRD Z144669/ 0002
9	Number of Scans	1
10	Receiver Gain	10.0
11	Relaxation Delay	0.1000
12	Pulse Width	9.5000
13	Presaturation Frequency	
14	Spectrometer Frequency	600.13
15	Spectral Width	8025.7
16	Lowest Frequency	-1121.6
17	Nucleus	1H
18	Acquired Size	48956
19	Spectral Size	65536
20	Digital Resolution	0.12

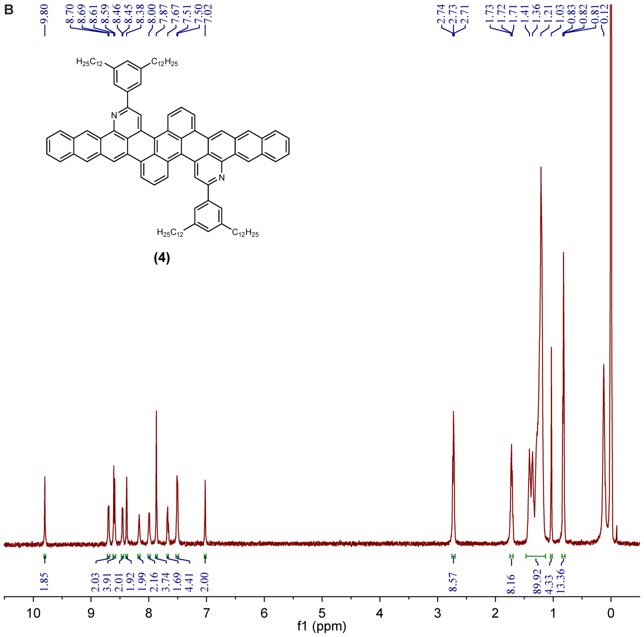

Parameter		Value
1	Comment	diluted some more 1H spectrum
2	Origin	Bruker BioSpin GmbH
3	Instrument	av600
4	Author	
5	Solvent	CS2
6	Temperature	298.0
7	Pulse Sequence	zg30
8	Experiment	1D
9	Probe	5 mm CPBBO BB-1H/ D Z-GRD Z144669/ 0002
10	Number of Scans	8
11	Receiver Gain	10.0
12	Relaxation Delay	0.1000
13	Pulse Width	9.5000
14	Presaturation Frequency	
15	Spectrometer Frequency	600.13
16	Spectral Width	8025.7
17	Lowest Frequency	-1127.1
18	Nucleus	1H
19	Acquired Size	48153
20	Spectral Size	65536

Supplementary Figure 17. (**A**) A representative ^{1}H NMR spectrum recorded for (**4**) after > 2 years of storage in the solid state. The concentration and conditions were comparable to those used for recording the spectra in Supplementary Figure 7. (**B**) Another representative ^{1}H NMR spectrum recorded for (**4**) after > 2 years of storage in the solid state. The concentration and conditions were comparable to those used for recording the spectra in Supplementary Figure 11A.

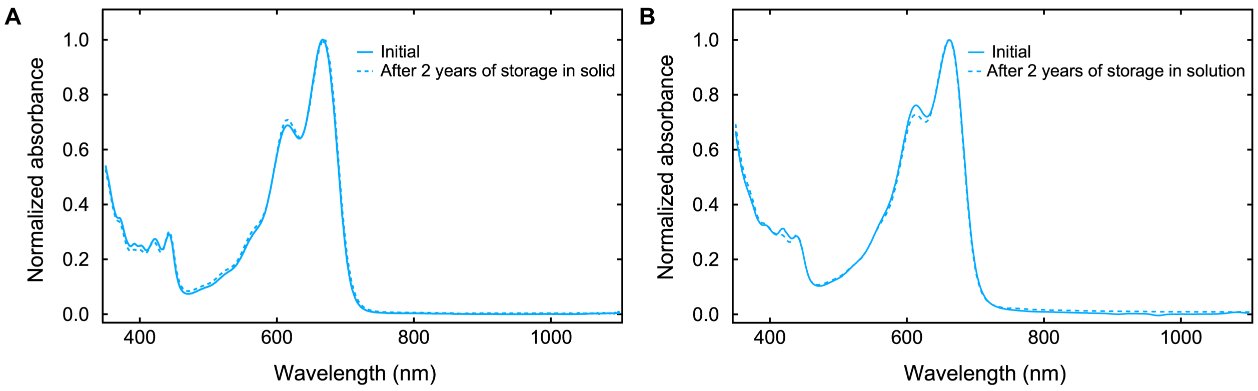

Supplementary Figure 18. (**A**) The experimental UV-Vis-NIR absorption spectra measured for TBN in chloroform shortly after initial preparation of the compound (blue solid line) and after > 2 years of storage in the solid state (blue dashed line). (**B**) The experimental UV-Vis-NIR absorption spectra measured for TBN in a 1:4 (v/v) mixture of Hi Sol 15 Aromatic 150 and VM&P Naphtha 66 solvents shortly after initial preparation of the compound (blue solid line) and after > 2 years of storage in solution (blue dashed line). Note that the absorption spectra were normalized to unity for clarity.

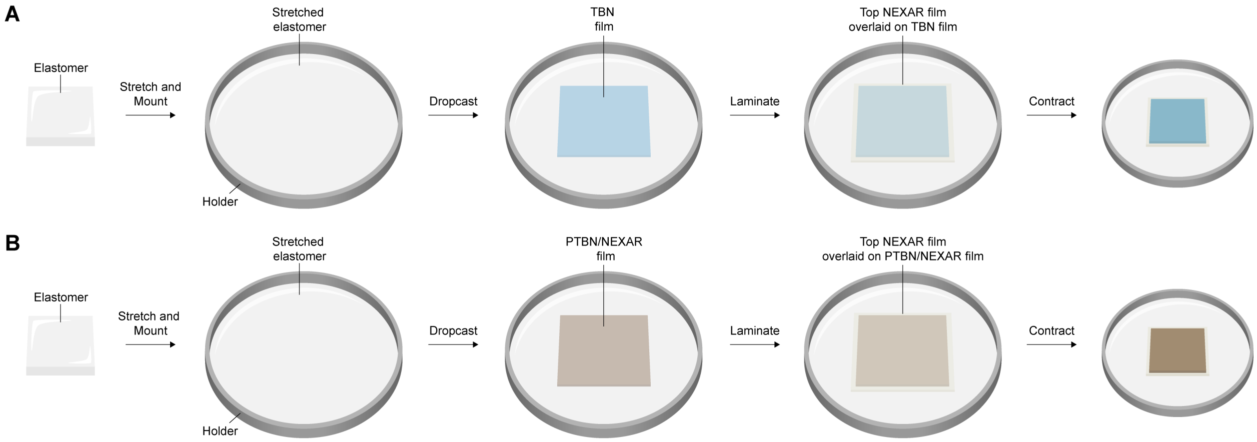

Supplementary Figure 19. (**A**) Schematic of the key steps required during the fabrication of tri-layer architectures with TBN-based central layers. (**B**) Schematic of the key steps required during the fabrication of tri-layer architectures with PTBN-based central layers.

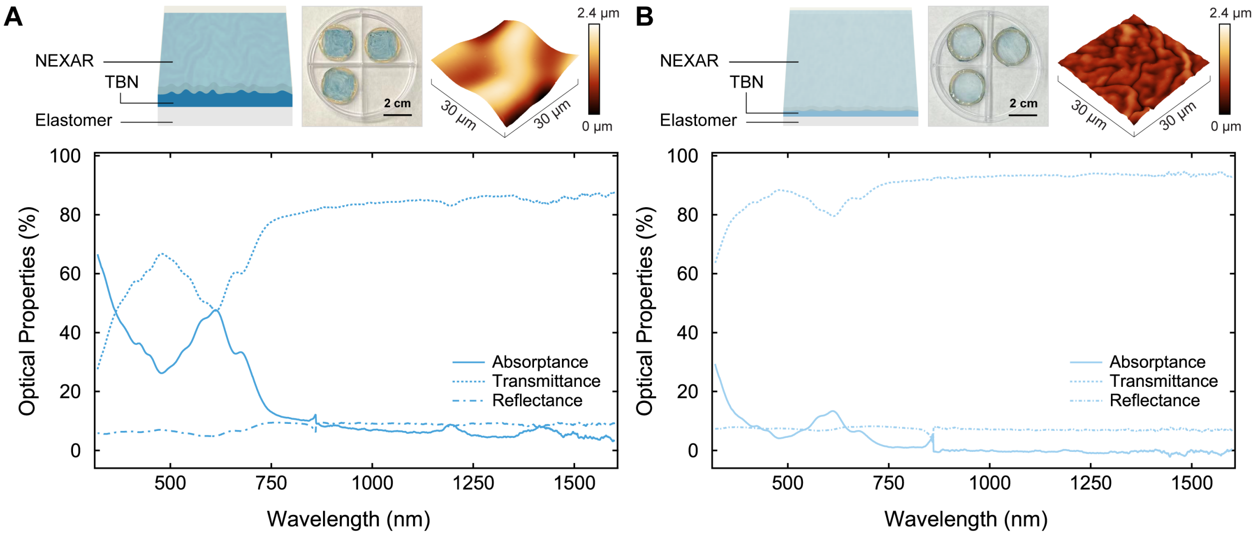

Supplementary Figure 20. (**A**) Top: Schematic (left), pictures (middle), and AFM images (right) of unactuated TBN-based tri-layer architectures fabricated from TBN solutions stored for > 2 years. Bottom: Representative UV-Vis-NIR absorptance (dark blue solid line), transmittance (dark blue dotted line), and reflectance (dark blue dashed-dotted line) spectra for unactuated tri-layer TBN-based architectures fabricated from TBN solutions stored for > 2 years. (**B**) Top: Schematic (left), pictures (middle), and AFM images (right) of actuated TBN-based tri-layer architectures fabricated from TBN solutions stored for > 2 years. Bottom: Representative UV-Vis-NIR absorptance (light blue solid line), transmittance (light blue dotted line), and reflectance (light blue dashed-dotted line) spectra for actuated TBN-based tri-layer architectures fabricated from TBN solutions stored for > 2 years.

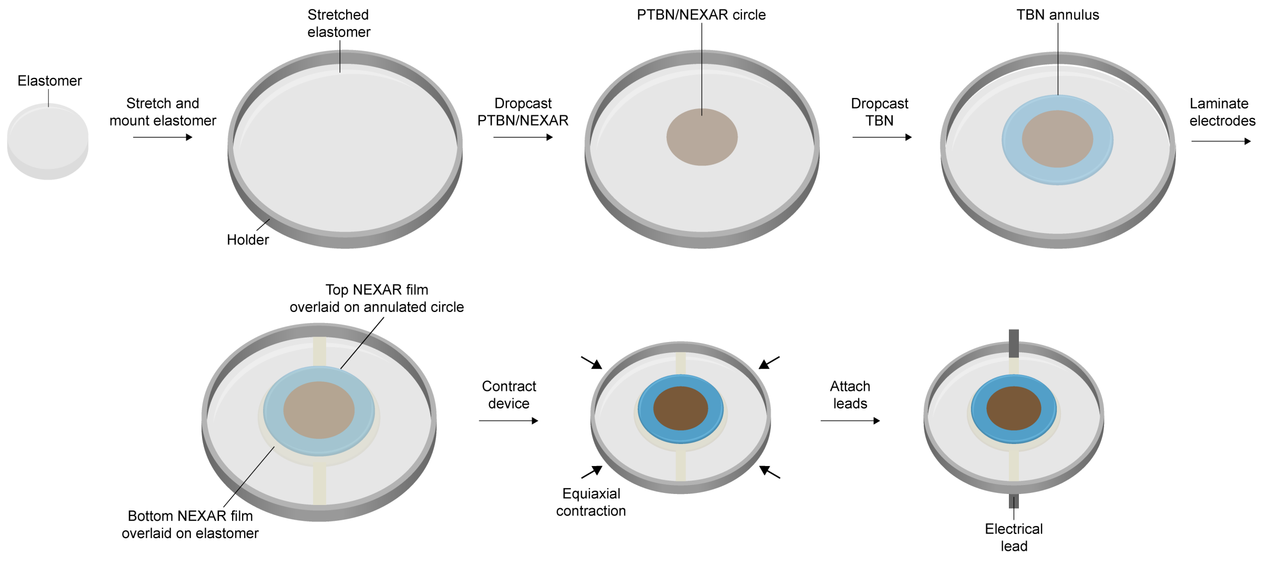

Supplementary Figure 21. Schematic of the key steps required during the fabrication of quad-layer devices for which the active layers consist of TBN-based blue annuli enclosing PTBN/NEXAR-based brown circles.

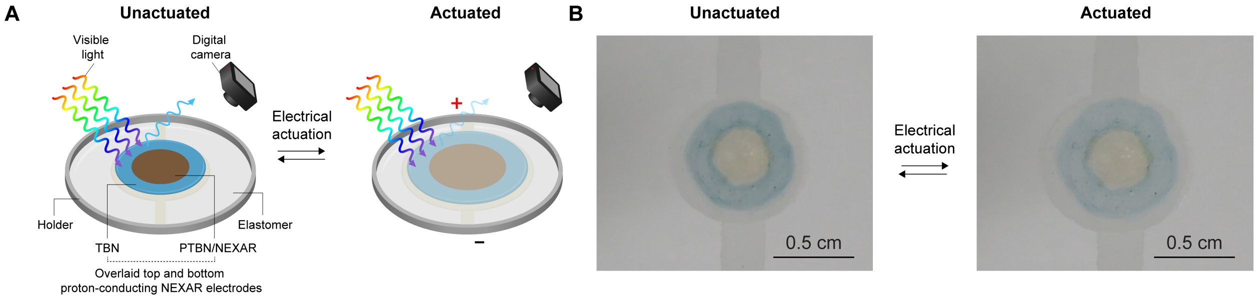

Supplementary Figure 22. (**A**) Schematic of a visible appearance-changing system undergoing monitoring during electrical actuation under standard indoor lighting in ambient conditions. (**B**) Representative digital camera images of an appearance-changing device before (left) and after (right) electrical actuation. The device was actuated with a bias of ~ 2.9 kV.

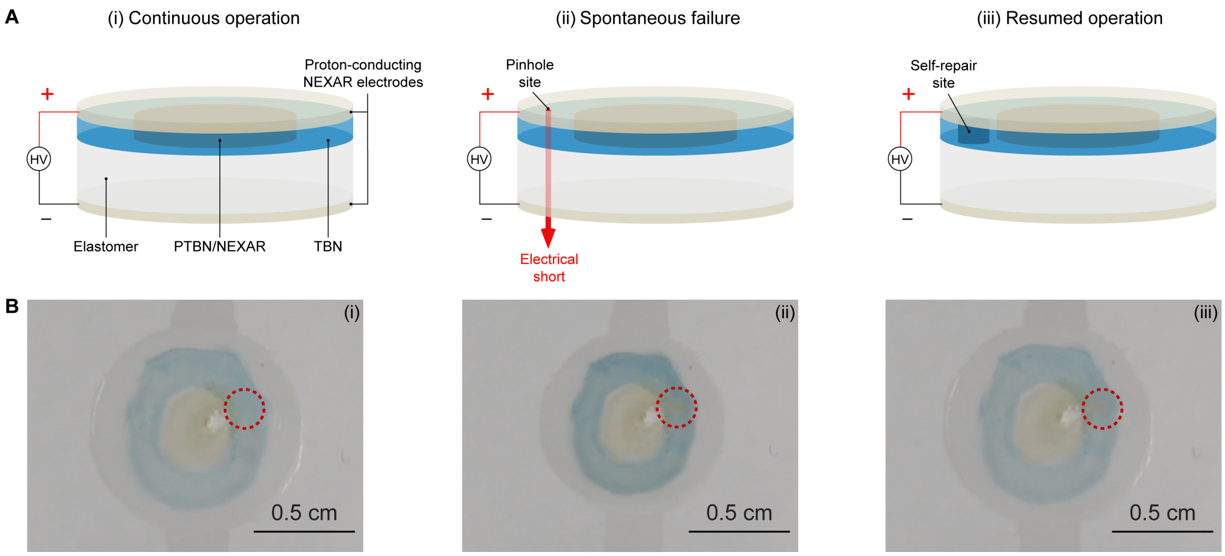

Supplementary Figure 23. (**A**) Schematic of a device that (i) initially continuously operates and features uniform coloration, (ii) spontaneously fails with a pause in function and acquires localized discoloration presumably due to a pinhole short, and (iii) resumes operation after autonomous self-repair with the localized discoloration remaining (from left to right). (**B**) Representative digital camera images of an appearance-changing device during the three stages shown in (A). Note that the breakdown region, which becomes and remains discolored, is indicated with red dashed circles. The device was actuated with a bias of ~ 2.9 kV.

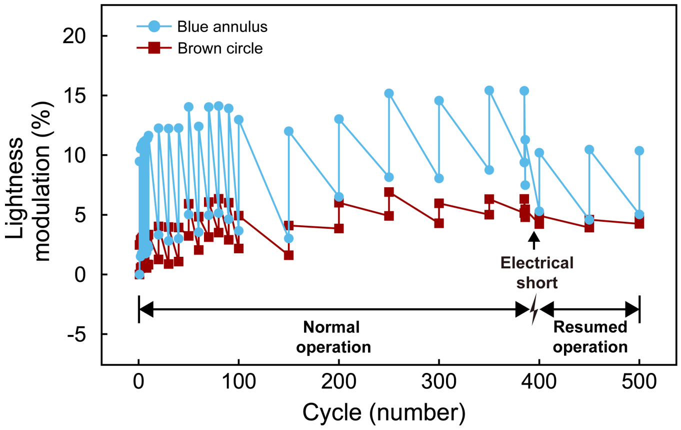

Supplementary Figure 24. Plot of the lightness modulation as a function of the actuation cycle number for a representative device's outer annulus (blue line) and inner circle (brown line) over 500 sequential actuation cycles. The device undergoes continuous operation with stable lightness modulation for nearly ~ 400 cycles, spontaneously fails with a corresponding brief pause in functionality, and resumes continuously operating with altered lightness modulation for ~ 100 more cycles.

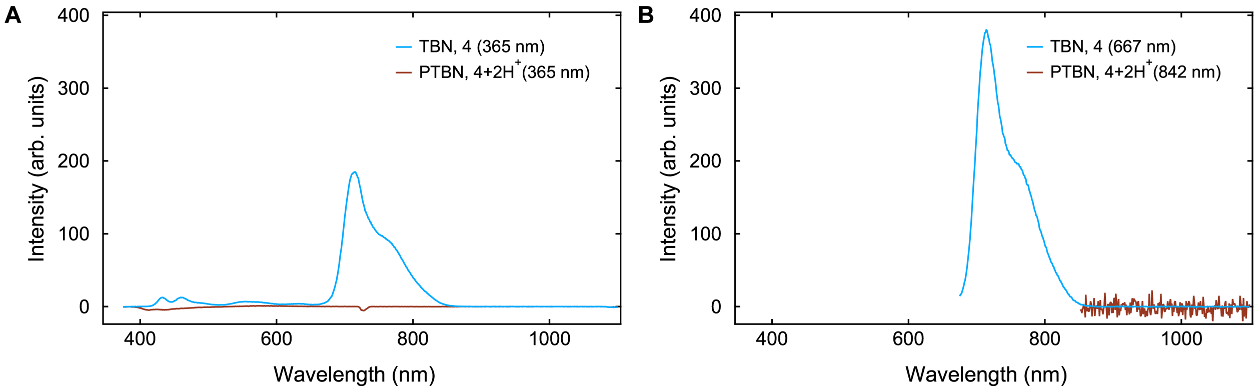

Supplementary Figure 25. (**A**) The fluorescence spectra measured for TBN (blue line) and PTBN (brown line) in chloroform at an identical excitation wavelength of 365 nm. (**B**) The fluorescence spectra measured for TBN (blue line) and PTBN (brown line) in chloroform at different excitation wavelengths of 667 nm and 842 nm, respectively.

III. Supplementary Tables

Material name	Chemical structure	Electronic configuration	Overall yield (%)	Synthesis method	Solubility	Stability when stored	Stability on a benchtop under irradiation	Thin-film processability	Device integration	Reference
**Surface-assisted or Solid-state approaches**										
Classic nonacene	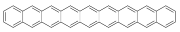	Open-shell	Not reported	Extreme temperature (30K), solid argon matrix	N/A	Not reported	Not reported	No	No	Tönshoff, 2010 [26]
		Closed-shell	Not reported	STM/AFM manipulation, ultra-high vacuum, Au(111) surface	N/A	Not reported	Not reported	No	No	Zuzak, 2017 [27]
		Open-shell	Not reported	STM/AFM manipulation, ultra-high vacuum, Au(111) surface	N/A	Not reported	Not reported	No	No	Urgel, 2019 [28]
		Not specified	Not reported	Photogeneration (470 nm), ultra-high vacuum, graphene/Ru(0001) surface	N/A	Not reported	Not reported	No	No	Ayani, 2021 [29]
**Solution-phase approaches**										
Arylthio-substituted nonacene	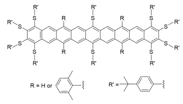	Closed-shell	~ 17	Standard solution-phase chemistry	CH_2Cl_2, $CDCl_3$	~ 24 hours in solution (in the dark) and ~ 6 weeks in the solid state (in the dark)	~ 2 hours in solution (exposed to air and light)	No	No	Kaur, 2010 [30]
Fluorine-substituted nonacene	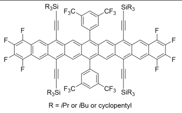	Open-shell	Not reported	Standard solution-phase chemistry	Toluene	> 2 days in the solid state (in the dark at 10°C)	< 6 hours in solution (exposed to air and bright laboratory light)	No	No	Purushothaman, 2011 [31]
Nonatwistacene	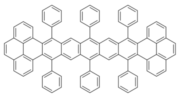	Not specified	Not reported	Standard solution-phase chemistry	CH_2Cl_2	~ 5 days in the solid state (exposed to air)	Not reported	No	No	Xiao, 2012 [11]
Oxygen-embedded quinoidal nonacene	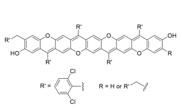	Closed-shell (open-shell species were formed upon one-electron oxidation)	~ 37	Standard solution-phase chemistry	THF, CH_2Cl_2, $CHCl_3$	Overnight in solution (under inert conditions, oxidized species) and several months in solution (exposed to air, neutral species)	Not reported	No	No	Wang, 2019 [12]
Butterfly nonacene	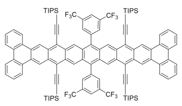	Closed-shell	~ 5	Standard solution-phase chemistry	n-hexane	~ 7 hours of half-life in solution (under N_2), ~ 6 weeks in the solid state (under N_2), and ~ 30 min half-life in solution (under ambient conditions)	Not reported	No	No	Muller, 2020 [13]
Peri-condensed nonacene	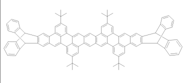	Not specified	~ 5	Standard solution-phase chemistry	$CHCl_3$	Not specified	Not specified	No	No	Yang, 2021 [32]
Classic nonacene	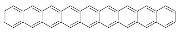	Not specified	Not reported	Standard solution-phase chemistry followed by heating in solid state or in high boiling point solvents	Highly insoluble	~ 2 months in the solid state (under dry argon, at room temperature) and thermally stable up to 450°C	Not reported	No	No	Jančařík, 2022 [33]
Nonacene-like molecule	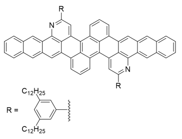	Closed-shell	~ 14	Standard solution-phase chemistry	$CHCl_3$, CS_2, THF, 1:4 (v/v) mixture of Hi Sol 15 Aromatic 150 and VM&P Naphtha 66	> 2 years in solution in a 1:4 (v/v) mixture of Hi Sol 15 Aromatic 150 and VM&P Naphtha 66 (in the dark, inside freezer) and > 2 years in the solid state (in the dark)	~ 40 hour half-life in solution (in protonated state, exposed to air and high-intensity light)	Yes	Yes	This work

Supplementary Table 1. A tabulated overview of the different nonacene variants prepared via surface-assisted or solid-state approaches and via solution-phase approaches, which summarizes the various reported molecules' key characteristics. Note the excellent solubility, exceptional stability, unique processability, and seminal device applications of the nonacene-like molecule described in this work.

Molecule	Transition/State	Dipole (*x*) (arb. units)	Dipole (*y*) (arb. units)	Dipole (*z*) (arb. units)	Total dipole squared	Oscillator strength	Reference
Nonacene	Higher energy transition (bright)	3.887 [a]	-	-	-	2.849 [b]	Chakraborty, 2013 [34] Bettinger, 2016 [35]
Nonacene	Lower energy transition (dark)	-	1.316 [a]	-	-	0.023 [b]	Chakraborty, 2013 [34] Bettinger, 2016 [35]
TBN	First excited state (bright)	5.1505	-0.7923	-0.0329	27.160	1.275	This work
TBN	Second excited state (dark)	0.0565	-0.0019	0.3631	0.1351	0.007	This work
PTBN	First excited state (bright)	5.6976	-0.8416	-0.0637	33.170	1.159	This work
PTBN	Second excited state (dark)	0.0701	0.0029	0.5132	0.268	0.011	This work

[a] Calculated using the Multireference Singles-Doubles Configuration Interaction method coupled with screened parameters in a Pariser-Parr-Pople model Hamiltonian.[34]
[b] Calculated using the Density Function Theory based Multireference Configuration Method.[35]

Supplementary Table 2. A comparison of the reported and calculated excited states, dipoles, and oscillator strengths for nonacene, TBN, and PTBN.

Device Number	Device Region	Lightness Modulation																			
		Before 1st cycle	After 1st cycle	Before 100th cycle	After 100th cycle	Before 150th cycle	After 150th cycle	Before 200th cycle	After 200th cycle	Before 250th cycle	After 250th cycle	Before 300th cycle	After 300th cycle	Before 350th cycle	After 350th cycle	Before 400th cycle	After 400th cycle	Before 450th cycle	After 450th cycle	Before 500th cycle	After 500th cycle
Device 1	Blue annulus	0%	8.1%	4.3%	14.1%	3.9%	14.2%	4.8%	14.1%	5.4%	14.4%	6.9%	15.7%	7.2%	15.5%	6.8%	15.0%	7.2%	15.1%	7.7%	15.7%
	Brown circle	0%	2.7%	2.9%	4.9%	3.0%	5.0%	3.2%	5.2%	3.6%	4.8%	4.6%	6.0%	4.4%	5.1%	3.9%	4.7%	3.6%	4.5%	4.0%	4.7%
Device 2	Blue annulus	0%	7.4%	4.8%	12.5%	4.1%	11.9%	4.6%	12.5%	5.0%	14.0%	4.8%	12.7%	5.2%	12.9%	5.3%	13.0%	6.2%	13.3%	6.0%	13.5%
	Brown circle	0%	2.8%	2.9%	5.4%	2.3%	4.7%	2.8%	5.1%	3.6%	6.0%	3.8%	4.7%	4.0%	4.9%	4.0%	4.8%	4.5%	4.8%	4.6%	5.3%
Device 3	Blue annulus	0%	6.8%	-4.5%	2.3%	2.8%	9.8%	5.1%	12.1%	5.5%	11.8%	6.2%	12.1%	6.3%	12.9%	6.0%	12.2%	6.8%	12.7%	7.1%	14.1%
	Brown circle	0%	3.1%	-4.0%	-1.9%	3.1%	5.5%	5.2%	8.1%	5.9%	8.7%	6.8%	8.2%	7.1%	9.1%	6.8%	8.5%	7.4%	8.7%	7.3%	8.2%
Device 4	Blue annulus	0%	7.6%	0.3%	8.2%	1.1%	9.1%	1.6%	9.7%	1.9%	10.6%	3.2%	12.0%	4.1%	13.2%	5.3%	13.6%	6.3%	14.4%	7.8%	16.2%
	Brown circle	0%	2.8%	-1.2%	1.0%	-0.4%	1.5%	-0.1%	-1.9%	0.1%	2.0%	0.7%	2.3%	0.9%	2.1%	1.0%	1.8%	1.6%	2.0%	2.5%	3.1%
Device 5	Blue annulus	0%	8.2%	3.5%	12.9%	4.4%	13.8%	4.4%	13.8%	6.5%	16.5%	7.3%	17.1%	7.7%	16.8%	10.1%	18.5%	11.0%	18.3%	11.7%	19.0%
	Brown circle	0%	1.7%	0.4%	2.2%	1.0%	2.8%	0.8%	2.4%	2.4%	3.9%	3.1%	4.0%	2.7%	3.2%	4.1%	4.3%	3.9%	4.1%	4.4%	4.6%
Device 6	Blue annulus	0%	6.9%	5.0%	13.4%	5.4%	13.9%	6.3%	15.6%	7.0%	15.9%	8.5%	16.4%	8.7%	16.2%	9.5%	16.4%	9.6%	16.1%	10.7%	17.1%
	Brown circle	0%	2.5%	2.7%	5.1%	2.8%	5.1%	3.5%	5.6%	3.2%	4.5%	3.1%	4.4%	3.3%	3.4%	3.4%	3.3%	3.2%	4.6%	4.2%	3.9%
Device 7	Blue annulus	0%	9.2%	3.4%	10.9%	4.6%	11.7%	5.1%	12.2%	6.1%	12.8%	6.0%	12.9%	6.2%	12.9%	6.4%	13.2%	6.3%	13.7%	6.9%	13.5%
	Brown circle	0%	2.9%	1.5%	4.9%	2.2%	4.6%	2.4%	4.5%	3.3%	4.8%	3.4%	4.5%	3.8%	4.5%	3.9%	4.4%	4.4%	4.8%	4.5%	4.6%
Device 8	Blue annulus	0%	6.2%	4.1%	12.3%	4.3%	12.4%	4.4%	12.8%	5.0%	13.0%	2.4%	4.5%	3.4%	6.4%	—	—	—	—	—	—
	Brown circle	0%	0.8%	0.9%	1.3%	1.1%	1.3%	1.2%	1.4%	1.5%	1.6%	0.5%	0.7%	1.1%	1.4%	—	—	—	—	—	—
Device 9	Blue annulus	0%	10.8%	6.4%	18.4%	4.4%	10.7%	5.0%	10.1%	—	—	—	—	—	—	—	—	—	—	—	—
	Brown circle	0%	3.2%	3.8%	7.1%	2.5%	4.4%	2.4%	4.2%	—	—	—	—	—	—	—	—	—	—	—	—
Device 10	Blue annulus	0%	9.5%	3.7%	13.0%	3.0%	12.0%	6.5%	13.0%	8.2%	15.2%	8.1%	14.6%	8.8%	15.4%	5.3%	10.2%	4.6%	10.5%	5.0%	10.4%
	Brown circle	0%	2.5%	2.2%	4.9%	1.6%	4.1%	3.8%	6.0%	4.9%	6.9%	4.3%	6.0%	5.0%	6.3%	4.2%	4.9%	3.9%	4.6%	4.2%	4.8%

Supplementary Table 3. The lightness modulation as a function of cycle number for 10 independent devices' blue annuli and brown circles over 500 sequential actuation cycles. Note that Devices 1-7 did not fail during cycling and that Devices 8-10 failed but then self-repaired during cycling. Device 1 corresponds to Figure 4E, Device 2 corresponds to Supplementary Figure 22, Device 9 corresponds to Supplementary Figure 23, and Device 10 corresponds to Supplementary Figure 24.

IV. Supplementary References

1. Park, Y. S., Dibble, D. J., Kim, J., Lopez, R. C., Vargas, E. & Gorodetsky, A. A. Synthesis of nitrogen-containing rubicene and tetrabenzopentacene derivatives. *Angew. Chem.* **128**, 3413–3416 (2016).
2. Umerani, M. J., Dibble, D. J., Wardrip, A. G., Mazaheripour, A., Vargas, E., Ziller, J. W. & Gorodetsky, A. A. Synthesis of polyquinolines via an AA/BB-type aza-Diels-Alder polymerization reaction. *J. Mater. Chem. C* **4**, 4060–4066 (2016).
3. Dibble, D. J. *et al.* Aza-Diels–Alder approach to diquinolineanthracene and polydiquinolineanthracene derivatives. *Org. Lett.* **20**, 502–505 (2018).
4. Feng, Z. *et al.* Bottom-up synthesis of nitrogen-containing graphene nanoribbons from the tetrabenzopentacene molecular motif. *Carbon* **170**, 677–684 (2020).
5. Frisch, M. J. *et al. Gaussian 16, Revision B.**01***. (2016) *Gaussian, Inc.*
6. Becke, A. D. Density-functional thermochemistry. III. The role of exact exchange. *J. Chem. Phys.* **98**, 5648–5652 (1993).
7. Lee, C., Yang, W. & Parr, R. G. Development of the Colle-Salvetti correlation-energy formula into a functional of the electron density. *Phys. Rev. B* **37**, 785–789 (1988).
8. Yang, Y., Davidson, E. R. & Yang, W. Nature of ground and electronic excited states of higher acenes. *Proc. Natl. Acad. Sci. U.S.A.* **113**, 5098–5107 (2016).
9. Fleischhauer, J., Zahn, S., Beckert, R., Grummt, U. W., Birckner, E. & Görls, H. A way to stable, highly emissive fluorubine dyes: tuning the electronic properties of azaderivatives of pentacene by introducing substituted pyrazines. *Chem. Eur. J.* **18**, 4549–4557 (2012).

10. Richards, G. J. *et al.* Amphiprotism-coupled near-infrared emission in extended pyrazinacenes containing seven linearly fused pyrazine units. *J. Am. Chem. Soc.* **141**, 19570–19574 (2019).

11. Xiao, J. *et al.* Synthesis and structure characterization of a stable nonatwistacene. *Angew. Chem. Int. Ed.* **51**, 6094–6098 (2012).

12. Wang, Y. *et al.* Synthesis and characterization of oxygen-embedded quinoidal pentacene and nonacene. *J. Am. Chem. Soc.* **141**, 2169–2176 (2019).

13. Müller, M. *et al.* Tetrabenzononacene: "butterfly wings" stabilize the core. *Angew. Chem.* **132**, 1982–1985 (2020).

14. Bendikov, M., Duong, H. M., Starkey, K., Houk, K. N., Carter, E. A. & Wudl, F. Oligoacenes: theoretical prediction of open-shell singlet diradical ground states. *J. Am. Chem. Soc.* **126**, 7416–7417 (2004).

15. Improta, R., Barone, V. & Santoro, F. Ab initio calculations of absorption spectra of large molecules in solution: Coumarin C153. *Angew. Chem. Int. Ed.* **46**, 405–408 (2007).

16. Santoro, F., Improta, R., Lami, A., Bloino, J. & Barone, V. Effective method to compute Franck-Condon integrals for optical spectra of large molecules in solution. *J. Chem. Phys.* **126**, 084509 (2007).

17. Muniz-Miranda, F., Pedone, A., Battistelli, G., Montalti, M., Bloino, J. & Barone, V. Benchmarking TD-DFT against vibrationally resolved absorption spectra at room temperature: 7-aminocoumarins as test cases. *J. Chem. Theory Comput.* **11**, 5371–5384 (2015).

18. Mukazhanova, A. *et al.* Accurate first-principles calculation of the vibronic spectrum of stacked perylene tetracarboxylic acid diimides. *J. Phys. Chem. A* **124**, 3055–3063 (2020).

19. Martin, R. L. Natural transition orbitals. *J. Chem. Phys.* **118**, 4775–4777 (2003).

20. Wu, C., Malinin, S. V., Tretiak, S. & Chernyak, V. Y. Multiscale modeling of electronic excitations in branched conjugated molecules using an exciton scattering approach. *Phys. Rev. Lett.* **100**, 057405 (2008).

21. Xu, C., Colorado Escobar, M. & Gorodetsky, A. A. Stretchable cephalopod-inspired multimodal camouflage systems. *Adv. Mater.* **32**, 1905717 (2020).

22. Liu, Y., Feng, Z., Xu, C., Chatterjee, A. & Gorodetsky, A. A. Reconfigurable micro-and nano-structured camouflage surfaces inspired by cephalopods. *ACS Nano* **15**, 17299–17309 (2021).

23. Hanssen, L., Kaplan, S. & Datla, R. Infrared Optical Properties of Materials. *NIST Spec. Publ.* 250–94 (National Institute of Standards and Technology, 2015).

24. Gilchrist, A. L. Lightness and brightness. *Curr. Biol.* **17**, R267–R269 (2007).

25. View Histograms and Pixel Values in *Adobe Photoshop Support*. https://helpx.adobe.com/photoshop/using/viewing-histograms-pixel-values.html (Adobe, 2020).

26. Tönshoff, C. & Bettinger, H. F. Photogeneration of octacene and nonacene. *Angew. Chem. Int. Ed.* **49**, 4125–4128 (2010).

27. Zuzak, R. *et al.* Nonacene generated by on-surface dehydrogenation. *ACS Nano* **11**, 9321–9329 (2017).

28. Urgel, J. I. *et al.* On-surface light-induced generation of higher acenes and elucidation of their open-shell character. *Nat. Commun.* **10**, 861 (2019).

29. Ayani, C. G. *et al.* Efficient photogeneration of nonacene on nanostructured graphene. *Nanoscale Horiz.* **6**, 744–750 (2021).

30. Kaur, I., Jazdzyk, M., Stein, N. N., Prusevich, P. & Miller, G. P. Design, synthesis, and characterization of a persistent nonacene derivative. *J. Am. Chem. Soc.* **132**, 1261–1263 (2010).

31. Purushothaman, B., Bruzek, M., Parkin, S. R., Miller, A. F. & Anthony, J. E. Synthesis and structural characterization of crystalline nonacenes. *Angew. Chem. Int. Ed.* **50**, 7013–7017 (2011).

32. Yang, X., Rominger, F. & Mastalerz, M. Benzo-fused perylene oligomers with up to 13 linearly annulated rings. *Angew. Chem. Int. Ed.* **60**, 7941–7946 (2021).

33. Jančařík, A., Holec, J., Nagata, Y., Šámal, M. & Gourdon, A. Preparative-scale synthesis of nonacene. *Nat. Commun.* **13**, 223 (2022).

34. Chakraborty, H. & Shukla, A. Pariser–Parr–Pople model based investigation of ground and low-lying excited states of long acenes. *J. Phys. Chem. A* **117**, 14220–14229 (2013).

35. Bettinger, H. F., Tonshoff, C., Doerr, M. & Sanchez-Garcia, E. Electronically excited states of higher acenes up to nonacene: A density functional theory/multireference configuration interaction study. *J. Chem. Theory. Comput.* **12**, 305–312 (2016).
