# Supplementary material for: Octopus-inspired deception and signaling systems from an exceptionally-stable acene variant
Source: Nat Commun. 2023 Dec 22;14:8528. doi: 10.1038/s41467-023-40163-7 (PMC10746719; doi:10.1038/s41467-023-40163-7)
Supplement: Supplementary file 2 — Description of Additional Supplementary Files [file 41467_2023_40163_MOESM2_ESM.pdf]

Description of Additional Supplementary Materials

Supplementary Data 1. The geometry calculated for TBN, **4**.

Supplementary Data 2. The geometry calculated for PTBN, **4+2H**$^{+}$.

Supplementary Movie 1. Representative video of a visible appearance-changing device during cyclical electrical actuation with a square waveform (frequency of 0.5 Hz, minima of 0 kV, and maxima of ~ 3.2 kV).

Supplementary Movie 2. Representative video of a near-infrared signature management device during cyclical electrical actuation with a square waveform (frequency of 0.5 Hz, minima of 0 kV, and maxima of ~ 3.2 kV).

Supplementary Movie 3. Representative video of a fluorescence signaling device during cyclical electrical actuation with a square waveform (frequency of 0.5 Hz, minima of 0 kV, and maxima of ~ 3.2 kV).
